# Supplementary material for: COVID-19 Vaccination Acceptance and Hesitancy in Healthcare Workers and the General Population: A Systematic Review and Policy Recommendations
Source: Int J Environ Res Public Health. 2024 Aug 28;21(9):1134. doi: 10.3390/ijerph21091134 (PMC11430955; doi:10.3390/ijerph21091134)
Supplement: Supplementary file 1 [file ijerph-21-01134-s001.zip › ijerph-3056496-supplementary.pdf]

Annex S1-Eligibility Criteria-PICO

Record ID

The study population is or includes Health care workers.	○Yes ○No<br>(Students are not participants)
The study addresses COVID-19 vaccination.	○Yes ○No
The study addresses HCW's requirements for vaccine acceptance by the population	○Yes ○No<br>(According to review question 1)
The study describes policies and strategies for vaccine acceptance by the population.	○ Yes ○ No<br>(According to review question 2)
The study describes approaches to improve vaccination teams´ quality and productivity.	○ Yes ○ No<br>(According to review question 3)
The study describes enablers and barriers for COVID-19 vaccination among Health care workers.	○ Yes ○ No<br>(According to review question 4)

Annex S2 – Search strategies per review question

DATABASE	SEARCH STRATEGIES – QUESTION 1<br>Q1 "What are countries' requirements to ensure COVID-19 vaccination coverage for the target population?"
PUBMED	((((COVID-19[mh] OR SARS-CoV-2[mh] OR Severe Acute Respiratory Syndrome Coronavirus 2[tiab] OR Coronavirus Disease 2019[tiab] OR 2019 Novel Coronavirus[tiab] OR 2019 New Coronavirus[tiab] OR Wuhan Coronavirus[tiab] OR COVID-19[tiab] OR SARS-CoV-2[tiab] OR 2019-nCoV[tiab] OR HCoV-19[tiab] OR nCoV-2019[tiab] OR Novel Coronavirus 2019-nCoV[tiab] OR Alpha Variant[tiab] OR Beta Variant[tiab] OR Gama Variant[tiab] OR Delta Variant[tiab] OR Delta Plus Variant[tiab] OR Omicron Variant[tiab] OR Lambda Variant[tiab]) AND (COVID-19 Vaccines[mh] OR Vaccination[mj] OR Vaccination Coverage[mj] OR Vaccination Coverage[tiab] OR Vaccination[tiab] OR Immunization[tiab] OR Vaccine*[tiab] OR Antivaccine[tiab] OR Anti-vaccine[tiab] OR Anti-vaccination[tiab] OR Coverage[ti] OR Infection*[ti])) AND (Vaccination Refusal[mh] OR Acceptance*[ti] OR Hesitanc*[ti] OR Refusal[ti] OR Acceptability[ti] OR Knowledge[ti] OR Attitude*[ti] OR Practice*[ti] OR Intention*[ti] OR Barrier*[ti] OR Enabler*[ti] OR Determinant*[ti])) AND (Health Workforce[mj] OR Workforce*[tiab] OR Health Manpower[tiab] OR Health Personnel[mj] OR Health Personnel*[tiab] OR Health Care Provider*[tiab] OR Healthcare Provider*[tiab] OR Health Care Worker*[tiab] OR Healthcare Worker*[tiab] OR Health Care Professional*[tiab] OR Healthcare Professional*[tiab] OR Human Resources for Health[tiab] OR Health Worker*[tiab] OR Allied Health Professional*[tiab] OR Healthcare Assistant*[tiab] OR Health Care Assistant*[tiab] OR Healthcare Support Worker*[tiab] OR Health Care Support Worker*[tiab] OR Caregivers[mj] OR

	Caregiver*[tiab] OR Licensed Practical Nurses[mj] OR Nursing Staff[mj] OR Nurses[mj] OR Nurse*[tiab] OR Nursing Personnel*[tiab] OR Nursing Staff*[tiab] OR Professional Nurse*[tiab] OR Nursing Associate*[tiab] OR Nursing Professional*[tiab] OR Nursing Assistant*[tiab] OR Auxiliary Nurse*[tiab] OR Nursing Auxiliar*[tiab] OR Licensed Practical Nurse*[tiab] OR Nursing Team*[tiab] OR Dentist*[tiab] OR Doctor*[tiab] OR Physicians[mj] OR Physician*[tiab] OR Pharmacist*[tiab] OR Physiotherapist*[tiab] OR Midwive*[tiab] OR Community Health Worker*[tiab] OR Community-Based Provider*[tiab] OR Laboratory Staff*[tiab] OR Paramedical Staff*[tiab] OR Paramedical Personnel*[tiab] OR Paramedic*[tiab])) NOT (Letter*[tw] OR Editorial*[tw] OR Release*[tw]) AND (English[lang] OR Portuguese[lang] OR Spanish[lang] OR French[lang] OR Italian[lang] OR Hindi[lang]) AND ("2020/01/01"[PDAT] : "2022/03/01"[PDAT])
**EMBASE**	('coronavirus disease 2019'/exp OR '2019 novel coronavirus disease':ti,ab OR '2019 novel coronavirus epidemic':ti,ab OR '2019 novel coronavirus infection':ti,ab OR '2019-ncov disease':ti,ab OR '2019-ncov infection':ti,ab OR 'COVID - 19':ti,ab OR 'COVID - 19 induced pneumonia':ti,ab OR 'covid 2019':ti,ab OR 'covid-19':ti,ab OR 'covid-19 induced pneumonia':ti,ab OR 'covid-19 pneumonia':ti,ab OR 'covid19':ti,ab OR 'sars coronavirus 2 infection':ti,ab OR 'sars coronavirus 2 pneumonia':ti,ab OR 'sars-cov-2 disease':ti,ab OR 'sars-cov-2 infection':ti,ab OR 'sars-cov-2 pneumonia':ti,ab OR 'sars-cov2 disease':ti,ab OR 'sars-cov2 infection':ti,ab OR 'sarscov2 disease':ti,ab OR 'sarscov2 infection':ti,ab OR 'wuhan coronavirus disease':ti,ab OR 'wuhan coronavirus infection':ti,ab OR 'coronavirus disease 2019':ti,ab OR 'coronavirus disease 2019 pneumonia':ti,ab OR 'coronavirus disease-19':ti,ab OR 'coronavirus infection 2019':ti,ab OR 'ncov 2019 disease':ti,ab OR 'ncov 2019 infection':ti,ab OR 'new coronavirus pneumonia':ti,ab OR 'novel coronavirus 2019 disease':ti,ab OR 'novel coronavirus 2019 infection':ti,ab OR 'novel coronavirus disease 2019':ti,ab OR 'novel coronavirus infected pneumonia':ti,ab OR 'novel coronavirus infection 2019':ti,ab OR 'novel coronavirus pneumonia':ti,ab OR 'paucisymptomatic coronavirus disease 2019':ti,ab OR 'severe acute respiratory syndrome 2':ti,ab OR 'severe acute respiratory syndrome 2 pneumonia':ti,ab OR 'severe acute respiratory syndrome cov-2 infection':ti,ab OR 'severe acute respiratory syndrome

	coronavirus 2 infection':ti,ab OR 'severe acute respiratory syndrome coronavirus 2019 infection':ti,ab OR 'Alpha Variant':ti,ab OR 'Beta Variant':ti,ab OR 'Gama Variant':ti,ab OR 'Delta Variant':ti,ab OR 'Delta Plus Variant':ti,ab OR 'Omicron Variant':ti,ab OR 'Lambda Variant':ti,ab) AND ('sars-cov-2 vaccine'/exp OR vaccine*:ti,ab OR vaccination:ti,ab OR immunization:ti,ab OR antivaccine:ti,ab OR 'anti vaccine':ti,ab) AND (acceptance:ti OR hesitancy:ti OR refusal:ti OR acceptability:ti OR knowledge:ti OR attitude*:ti OR practice*:ti OR intention*:ti OR barrier*:ti OR enabler*:ti OR determinant*:ti) AND ('health workforce'/mj OR 'health care labour force':ti,ab OR 'health care manpower':ti,ab OR 'health care work force':ti,ab OR 'health care workforce':ti,ab OR 'health labor force':ti,ab OR 'health labour force':ti,ab OR 'health manpower':ti,ab OR 'health work force':ti,ab OR 'health workforce':ti,ab OR 'healthcare labor force':ti,ab OR 'healthcare labour force':ti,ab OR 'healthcare manpower':ti,ab OR 'healthcare work force':ti,ab OR 'healthcare workforce':ti,ab OR 'health care labor force':ti,ab OR 'health care personnel'/mj OR 'health care personnel*':ti,ab OR 'health care practitioner*':ti,ab OR 'health care professional*':ti,ab OR 'health care provider*':ti,ab OR 'health care worker*':ti,ab OR 'health personnel*':ti,ab OR 'health worker*':ti,ab OR 'healthcare personnel':ti,ab OR 'healthcare practitioner':ti,ab OR 'healthcare professional':ti,ab OR 'healthcare provider':ti,ab OR 'healthcare worker*':ti,ab OR 'caregiver'/mj OR 'caregiver*':ti,ab OR 'human resources for health':ti,ab OR 'licensed practical nurse'/mj OR 'licensed practical nurse*':ti,ab OR 'licensed vocational nurse':ti,ab OR 'nursing staff'/mj OR 'hospital nursing staff':ti,ab OR 'nurse staffing':ti,ab OR 'nursing manpower':ti,ab OR 'nursing personnel':ti,ab OR 'nursing staff':ti,ab OR 'nurse'/mj OR 'nurse*':ti,ab OR 'nursing associate*':ti,ab OR 'nursing assistant'/mj OR 'nursing assistant*':ti,ab OR 'auxiliary nurse*':ti,ab OR 'nursing auxiliar*':ti,ab OR 'team nursing'/mj OR 'team nursing':ti,ab OR 'dentist'/mj OR 'dentist*':ti,ab OR 'physician'/mj OR 'doctor*':ti,ab OR 'physician*':ti,ab OR 'private physician':ti,ab OR 'pharmacist'/mj OR 'pharmacist*':ti,ab OR 'physiotherapist'/mj OR 'physical therapist*':ti,ab OR 'physiotherapist*':ti,ab OR 'midwife'/mj OR 'midwife':ti,ab OR 'midwifery':ti,ab OR 'midwives':ti,ab OR 'health auxiliary'/mj OR 'auxiliary health worker':ti,ab OR 'community health worker*':ti,ab OR 'medical auxiliary':ti,ab OR 'community-based provider*':ti,ab OR 'laboratory staff*':ti,ab OR 'paramedical

	personnel'/mj OR 'healthcare assistant':ti,ab OR 'healthcare support worker':ti,ab OR 'para medical personnel':ti,ab OR 'paramedical personnel':ti,ab OR 'paramedical professional':ti,ab OR 'paramedical staff':ti,ab) NOT ('letter*':ti,ab OR 'editorial*':ti,ab OR 'release*':ti,ab) AND ([english]/lim OR [french]/lim OR [hindi]/lim OR [italian]/lim OR [portuguese]/lim OR [spanish]/lim) AND [01-01-2020]/sd NOT [01-03-2022]/sd AND [embase]/lim NOT ([embase]/lim AND [medline]/lim)
**SCOPUS**	ALL(COVID-19 OR SARS-CoV-2 OR "Severe Acute Respiratory Syndrome Coronavirus 2" OR "Coronavirus Disease 2019" OR "2019 Novel Coronavirus" OR "2019 New Coronavirus" OR "Wuhan Coronavirus" OR 2019-nCoV OR HCoV-19 OR nCoV-2019 OR "Novel Coronavirus 2019-nCoV" OR "Alpha Variant" OR "Beta Variant" OR "Gama Variant" OR "Delta Variant" OR "Delta Plus Variant" OR "Omicron Variant" OR "Lambda Variant") AND ALL("COVID-19 Vaccines" OR Vaccination OR "Vaccination Coverage" OR "Vaccination Coverage" OR Vaccination OR Immunization OR Vaccine* OR autovaccine OR Anti-vaccine OR Anti-vaccination OR Coverage OR Infection*) AND TITLE(Acceptance* OR Hesitanc* OR Refusal OR Acceptability OR Knowledge OR Attitude* OR Practice* OR Intention* OR Barrier* OR Enabler* OR Determinant*) AND TITLE("Health Workforce" OR "Health Workforces" OR "Health Manpower" OR "Health Personnel" OR "Health Personnels" OR "Health Care Providers" OR "Healthcare Providers" OR "Health Care Workers" OR "Healthcare Workers" OR "Health Care Professionals" OR "Healthcare Professionals" OR Caregiver* OR "Licensed Practical Nurses" OR "Nursing Staff" OR Nurse* OR "Nursing Personnel" OR "Professional Nurses" OR "Nursing Associate" OR "Nursing Professionals" OR "Nursing Assistant" OR "Auxiliary Nurses" OR "Nursing Auxiliary" OR "Licensed Practical Nurses" OR "Nursing Team" OR Dentists OR Doctors OR Physicians OR Pharmacists OR Physiotherapists OR Midwives OR "Community Health Workers" OR "Community-Based Providers" OR "Laboratory Staff" OR "Paramedical Staff" OR "Paramedical Personnel") AND (LIMIT-TO(DOCTYPE, "ar")) AND (LIMIT-TO(LANGUAGE, "English") OR LIMIT-TO(LANGUAGE, "Spanish") OR LIMIT-TO(LANGUAGE, "French") OR LIMIT-TO(LANGUAGE, "Portuguese")) OR LIMIT-

	TO(LANGUAGE, "Italian")) OR LIMIT-TO(LANGUAGE, "Hindi")) AND (LIMIT-TO(PUBYEAR, 2022) OR LIMIT-TO(PUBYEAR, 2021) OR LIMIT-TO(PUBYEAR, 2020))
**BVS/LILACS**	(covid-19 OR sars-cov-2 OR "Severe Acute Respiratory Syndrome Coronavirus 2" OR "Coronavirus Disease 2019" OR "2019 Novel Coronavirus" OR "2019 New Coronavirus" OR "Wuhan Coronavirus" OR 2019-ncov OR hcov-19 OR ncov-2019 OR "Novel Coronavirus 2019-nCoV" OR "Alpha Variant" OR "Beta Variant" OR "Gama Variant" OR "Delta Variant" OR "Delta Plus Variant" OR "Omicron Variant" OR "Lambda Variant") AND ("COVID-19 Vaccines" OR vaccination OR "Vaccination Coverage" OR "Vaccination Coverage" OR vaccination OR immunization OR vaccine* OR antivaccine OR anti-vaccine OR anti-vaccination OR coverage OR vacina* OR anti-vacina OR imunização OR cobertura* OR vacuna* OR antivacuna OR inmunización) AND (acceptance* OR hesitancy OR refusal OR acceptability OR knowledge OR attitude* OR practice* OR intention* OR barrier* OR enabler* OR determinant* OR aceitação* OR hesitação OR rejeição OR recusa OR aceitabilidade OR conhecimento OR atitude* OR prática* OR intenção OR barreira* OR facilitador* OR determinante* OR aceptación* OR vacilación OR rechazo OR aceptabilidad OR conocimiento OR actitud* OR práctica* OR intención* OR barrera* OR facilitador*) AND ("Health Workforce" OR "Health Workforces" OR "Health Manpower" OR "Health Personnel" OR "Health Personnels" OR "Health Care Providers" OR "Healthcare Providers" OR "Health Care Workers" OR "Healthcare Workers" OR "Health Care Professionals" OR "Healthcare Professionals" OR caregiver* OR "Licensed Practical Nurses" OR "Nursing Staff" OR Nurse* OR "Nursing Personnel" OR "Professional Nurses" OR "Nursing Associate" OR "Nursing Professionals" OR "Nursing Assistant" OR "Auxiliary Nurses" OR "Nursing Auxiliary" OR "Licensed Practical Nurses" OR "Nursing Team" OR Dentist* OR Doctor* OR Physicians OR Pharmacist* OR Physiotherapist* OR Midwive* OR "Community Health Workers" OR "Community-Based Providers" OR "Laboratory Staff" OR Paramedical OR "força de trabalho em saúde" OR "recursos humanos em saúde" OR "pessoal de saúde" OR "provedores de saúde" OR "trabalhadores em saúde" OR "profissionais de saúde" OR cuidador* OR enfermeir* OR "equipe de enfermagem" OR "pessoal de enfermagem" OR

	"profissionais de enfermagem" OR "assistente de enfermagem" OR "auxiliares de enfermagem" OR dentista* OR médico* OR farmacêutico* OR fisioterapeuta* OR parteira* OR "trabalhadores comunitários de saúde" OR "provedores de base comunitária" OR "equipe de laboratório" OR paramédico* OR "personal de salud" OR "recursos humanos en salud" OR "proveedores de salud" OR "trabajadores de la salud" OR "servicios profesionales de salud" OR "personal de enfermería" OR enfermera* OR "personal de enfermería" OR "profesionales de enfermería" OR "auxiliar de enfermería" OR "auxiliares de enfermería" OR partera* OR "trabajadores de salud comunitarios" OR "proveedores comunitarios" OR "personal de laboratorio") AND (db:("LILACS")) AND (year_cluster:[2020 TO 2022])
**CINAHL**	(COVID-19 OR SARS-CoV-2 OR "Severe Acute Respiratory Syndrome Coronavirus 2" OR "Coronavirus Disease 2019" OR "2019 Novel Coronavirus" OR "2019 New Coronavirus" OR "Wuhan Coronavirus" OR 2019-nCoV OR HCoV-19 OR nCoV-2019 OR "Novel Coronavirus 2019-nCoV" OR "Alpha Variant" OR "Beta Variant" OR "Gama Variant" OR "Delta Variant" OR "Delta Plus Variant" OR "Omicron Variant" OR "Lambda Variant") AND ("COVID-19 Vaccines" OR Vaccination OR "Vaccination Coverage" OR "Vaccination Coverage" OR Vaccination OR Immunization OR Vaccine* OR Antivaccine OR Anti-vaccine OR Anti-vaccination OR Coverage OR Infection*) AND TI(Acceptance* OR Hesitanc* OR Refusal OR Acceptability OR Knowledge OR Attitude* OR Practice* OR Intention* OR Barrier* OR Enabler* OR Determinant*) AND ("Health Workforce" OR "Health Workforces" OR "Health Manpower" OR "Health Personnel" OR "Health Personnels" OR "Health Care Providers" OR "Healthcare Providers" OR "Health Care Workers" OR "Healthcare Workers" OR "Health Care Professionals" OR "Healthcare Professionals" OR Caregiver* OR "Licensed Practical Nurses" OR "Nursing Staff" OR Nurse* OR "Nursing Personnel" OR "Professional Nurses" OR "Nursing Associate" OR "Nursing Professionals" OR "Nursing Assistant" OR "Auxiliary Nurses" OR "Nursing Auxiliary" OR "Licensed Practical Nurses" OR "Nursing Team" OR Dentists OR Doctors OR Physicians OR Pharmacists OR Physiotherapists OR Midwives OR "Community Health

	Workers" OR "Community-Based Providers" OR "Laboratory Staff" OR "Paramedical Staff" OR "Paramedical Personnel") AND (LA English OR LA Portuguese OR LA Spanish OR LA French OR LA Italian OR LA Hindi) AND (PY 2020 OR PY 2021 OR PY 2022)
**WHO COVID-19**	(ti:(covid-19 OR sars-cov-2 OR "Severe Acute Respiratory Syndrome Coronavirus 2" OR "Coronavirus Disease 2019" OR "2019 Novel Coronavirus" OR "2019 New Coronavirus" OR "Wuhan Coronavirus" OR 2019-ncov OR hcov-19 OR ncov-2019 OR "Novel Coronavirus 2019-nCoV" OR "Alpha Variant" OR "Beta Variant" OR "Gama Variant" OR "Delta Variant" OR "Delta Plus Variant" OR "Omicron Variant" OR "Lambda Variant")) AND (ti:("Health Workforce" OR "Health Workforces" OR "Health Manpower" OR "Health Personnel" OR "Health Personnels" OR "Health Care Providers" OR "Healthcare Providers" OR "Health Care Workers" OR "Healthcare Workers" OR "Health Care Professionals" OR "Healthcare Professionals" OR caregiver* OR "Licensed Practical Nurses" OR "Nursing Staff" OR nurse* OR "Nursing Personnel" OR "Professional Nurses" OR "Nursing Associate" OR "Nursing Professionals" OR "Nursing Assistant" OR "Auxiliary Nurses" OR "Nursing Auxiliary" OR "Licensed Practical Nurses" OR "Nursing Team" OR dentist* OR doctor* OR physicians OR pharmacist* OR physiotherapist* OR midwive* OR "Community Health Workers" OR "Community-Based Providers" OR "Laboratory Staff" OR paramedical OR "força de trabalho em saúde" OR "recursos humanos em saúde" OR "pessoal de saúde" OR "provedores de saúde" OR "trabalhadores em saúde" OR "profissionais de saúde" OR cuidador* OR enfermeir* OR "equipe de enfermagem" OR "pessoal de enfermagem" OR "profissionais de enfermagem" OR "assistente de enfermagem" OR "auxiliares de enfermagem" OR dentista* OR médico* OR farmacêutico* OR fisioterapeuta* OR parteira* OR "trabalhadores comunitários de saúde" OR "provedores de base comunitária" OR "equipe de laboratório" OR paramédico* OR "personal de salud" OR "recursos humanos en salud" OR "proveedores de salud" OR "trabajadores de la salud" OR "servicios profesionales de salud" OR "personal de enfermería" OR enfermera* OR "personal de enfermería" OR "profesionales de enfermería" OR "auxiliar de enfermería" OR "auxiliares de enfermería" OR partera* OR "trabajadores de salud comunitarios" OR "proveedores comunitarios" OR "personal de laboratorio")) AND db:("GREY-COVIDWHO" OR "WHOIRIS")

**GOOGLE SCHOLAR**	allintitle: ("COVID-19 Vaccines" OR "COVID-19 Vaccination" OR "COVID-19 Vaccine") AND ("Health Workforce" OR "Health Personnel" OR "Health Personnels" OR "Healthcare Workers" OR "Healthcare Professionals")

**DATABASE**	**SEARCH STRATEGIES – QUESTION 2** Q2"How are countries managing COVID-19 policy, regulation, prioritization, and mandatory vaccination of the target population?"
**PUBMED**	(((COVID-19[mj] OR SARS-CoV-2[mj] OR Severe Acute Respiratory Syndrome Coronavirus 2[tiab] OR Coronavirus Disease 2019[tiab] OR 2019 Novel Coronavirus[tiab] OR 2019 New Coronavirus[tiab] OR Wuhan Coronavirus[tiab] OR COVID-19[tiab] OR SARS-CoV-2[tiab] OR 2019-nCoV[tiab] OR HCoV-19[tiab] OR nCoV-2019[tiab] OR Novel Coronavirus 2019-nCoV[tiab] OR Alpha Variant[tiab] OR Beta Variant[tiab] OR Gamma Variant[tiab] OR Delta Variant[tiab] OR Delta Plus Variant[tiab] OR Omicron Variant[tiab] OR Lambda Variant[tiab]) AND (Immunization Programs[mj] OR Immunization Program*[tiab] OR Mass Vaccination[mj] OR Mass Vaccination*[tiab] OR Mass Immunization*[tiab] OR Vaccination Programm*[tiab] OR Vaccine Programm*[tiab] OR Vaccination Team*[tiab] OR Vaccine Team*[tiab] OR Vaccination Campaign*[tiab] OR Vaccine Campaign*[tiab] OR Vaccination*[ti] OR Anti-vaccine[ti] OR Anti-vaccination[ti])) AND (Acceptance*[ti] OR Hesitanc*[ti] OR Refusal[ti] OR Acceptability[ti] OR Barrier*[ti] OR Enabler*[ti] OR Challenge*[ti] OR Facilitator*[ti] OR Performance*[ti] OR Improvement*[ti] OR Optimizer*[ti] OR Strateg*[ti] OR Mechanism*[ti] OR Tool*[ti] OR Productivit*[ti] OR Success*[ti])) NOT (Letter*[tw] OR Editorial*[tw] OR Release*[tw]) AND

	(English[lang] OR Portuguese[lang] OR Spanish[lang] OR French[lang] OR Italian[lang] OR Hindi[lang]) AND ("2020/01/01"[PDAT] : "2022/03/01"[PDAT])
**EMBASE**	('coronavirus disease 2019'/mj OR '2019 novel coronavirus disease':ti,ab OR '2019 novel coronavirus epidemic':ti,ab OR '2019 novel coronavirus infection':ti,ab OR '2019-ncov disease':ti,ab OR '2019-ncov infection':ti,ab OR 'COVID - 19':ti,ab OR 'COVID - 19 induced pneumonia':ti,ab OR 'covid 2019':ti,ab OR 'covid-19':ti,ab OR 'covid-19 induced pneumonia':ti,ab OR 'covid-19 pneumonia':ti,ab OR 'covid19':ti,ab OR 'sars coronavirus 2 infection':ti,ab OR 'sars coronavirus 2 pneumonia':ti,ab OR 'sars-cov-2 disease':ti,ab OR 'sars-cov-2 infection':ti,ab OR 'sars-cov-2 pneumonia':ti,ab OR 'sars-cov2 disease':ti,ab OR 'sars-cov2 infection':ti,ab OR 'sarscov2 disease':ti,ab OR 'sarscov2 infection':ti,ab OR 'wuhan coronavirus disease':ti,ab OR 'wuhan coronavirus infection':ti,ab OR 'coronavirus disease 2019':ti,ab OR 'coronavirus disease 2019 pneumonia':ti,ab OR 'coronavirus disease-19':ti,ab OR 'coronavirus infection 2019':ti,ab OR 'ncov 2019 disease':ti,ab OR 'ncov 2019 infection':ti,ab OR 'new coronavirus pneumonia':ti,ab OR 'novel coronavirus 2019 disease':ti,ab OR 'novel coronavirus 2019 infection':ti,ab OR 'novel coronavirus disease 2019':ti,ab OR 'novel coronavirus infected pneumonia':ti,ab OR 'novel coronavirus infection 2019':ti,ab OR 'novel coronavirus pneumonia':ti,ab OR 'paucisymptomatic coronavirus disease 2019':ti,ab OR 'severe acute respiratory syndrome 2':ti,ab OR 'severe acute respiratory syndrome 2 pneumonia':ti,ab OR 'severe acute respiratory syndrome cov-2 infection':ti,ab OR 'severe acute respiratory syndrome coronavirus 2 infection':ti,ab OR 'severe acute respiratory syndrome coronavirus 2019 infection':ti,ab OR 'Alpha Variant':ti,ab OR 'Beta Variant':ti,ab OR 'Gama Variant':ti,ab OR 'Delta Variant':ti,ab OR 'Delta Plus Variant':ti,ab OR 'Omicron Variant':ti,ab OR 'Lambda Variant':ti,ab) AND ('immunization program*':ti,ab OR 'mass immunization'/mj OR 'mass immunisation' OR 'mass immunization' OR 'mass vaccination' OR 'vaccination'/mj OR 'vaccination program':ti,ab OR 'vaccination programme':ti,ab OR 'vaccine programm*':ti,ab OR 'vaccination team*':ti,ab OR 'vaccine team*':ti,ab OR 'vaccination campaign*':ti,ab OR 'vaccine campaign*':ti,ab OR vaccination*:ti OR 'anti vaccine':ti OR 'anti vaccination*':ti) AND ('vaccination refusal'/mj OR 'refusal of vaccination':ti OR 'vaccination refusal':ti OR 'vaccine refusal':ti OR acceptance:ti OR

	hesitancy:ti OR refusal:ti OR acceptability:ti OR intention*:ti OR barrier*:ti OR enabler*:ti OR determinant*:ti OR facilitator*:ti OR performance*:ti OR improvement*:ti OR optimizer*:ti OR strateg*:ti OR mechanism*:ti OR productivit*:ti OR tool*:ti OR success*:ti) AND ([english]/lim OR [french]/lim OR [hindi]/lim OR [italian]/lim OR [portuguese]/lim OR [spanish]/lim) AND [01-01-2020]/sd NOT [03-01-2022]/sd AND [embase]/lim NOT ([embase]/lim AND [medline]/lim)
**SCOPUS**	TITLE(COVID-19 OR SARS-CoV-2 OR "Severe Acute Respiratory Syndrome Coronavirus 2" OR "Coronavirus Disease 2019" OR "2019 Novel Coronavirus" OR "2019 New Coronavirus" OR "Wuhan Coronavirus" OR 2019-nCoV OR HCoV-19 OR nCoV-2019 OR "Novel Coronavirus 2019-nCoV" OR "Alpha Variant" OR "Beta Variant" OR "Gamma Variant" OR "Delta Variant" OR "Delta Plus Variant" OR "Omicron Variant" OR "Lambda Variant") AND TITLE("Immunization Program" OR "Immunization Programs" OR "Mass Vaccination" OR "Mass Immunization" OR "Vaccination Programme" OR "Vaccination Programmes" OR "Vaccine Programme" OR "Vaccine Programmes" OR "Vaccination Team" OR "Vaccination Teams" OR "Vaccine Team" OR "Vaccine Teams" OR "Vaccination Campaign" OR "Vaccination Campaignes" OR "Vaccine Campaigne" OR "Vaccine Campaignes" OR Vaccination OR Immunization OR Antivaccine OR Anti-Vaccine OR Anti-Vaccination) AND TITLE(Refusal OR Acceptance* OR Hesitancy OR Acceptability OR Intention* OR Barrier* OR Enabler* OR Facilitator* OR Performance* OR Improvement* OR Optimizer* OR Strateg* OR Productivit* OR Mechanism* OR Productivity* OR Tool* OR Success*) AND (LIMIT-TO(DOCTYPE, "ar")) AND (LIMIT-TO(LANGUAGE, "English") OR LIMIT-TO(LANGUAGE, "Spanish") OR LIMIT-TO(LANGUAGE, "French") OR LIMIT-TO(LANGUAGE, "Portuguese")) OR LIMIT-TO(LANGUAGE, "Italian")) OR LIMIT-TO(LANGUAGE, "Hindi")) AND (LIMIT-TO(PUBYEAR, 2022) OR LIMIT-TO(PUBYEAR, 2021) OR LIMIT-TO(PUBYEAR, 2020))

**LILACS**	(covid-19 OR sars-cov-2 OR "Severe Acute Respiratory Syndrome Coronavirus 2" OR "Coronavirus Disease 2019" OR "2019 Novel Coronavirus" OR "2019 New Coronavirus" OR "Wuhan Coronavirus" OR 2019-ncov OR hcov-19 OR ncov-2019 OR "Novel Coronavirus 2019-nCoV" OR "Alpha Variant" OR "Beta Variant" OR "Gamma Variant" OR "Delta Variant" OR "Delta Plus Variant" OR "Omicron Variant" OR "Lambda Variant") AND ("Immunization Program" OR "Immunization Programs" OR "Mass Vaccination" OR "Mass Immunization" OR "Vaccination Programme" OR "Vaccination Programmes" OR "Vaccine Programme" OR "Vaccine Programmes" OR "Vaccination Team" OR "Vaccination Teams" OR "Vaccine Team" OR "Vaccine Teams" OR "Vaccination Campaign" OR "Vaccination Campaignes" OR "Vaccine Campaigne" OR "Vaccine Campaignes" OR vaccination OR immunization OR antivaccine OR anti-vaccine OR anti-vaccination OR "Programa de imunização" OR "Programas de imunização" OR "Vacinação em massa" OR "Imunização em massa" OR "Programa de vacinação" OR "Programas de vacinação" OR "Equipe de vacinação" OR "Equipes de vacinação" OR "Campanha de vacinação" OR "Campanhas de vacinação" OR anti-vacina OR imunização OR "Programa de vacunación" OR "Programas de vacunación" OR "Inmunización masiva" OR "Inmunizaciónes masivas" OR "Equipo de vacunación" OR "Equipos de vacunación" OR "Campaña de vacunación" OR "Campañas de vacunación" OR antivacuna OR inmunización) AND ti:(refusal OR acceptance* OR hesitancy OR refusal OR acceptability OR intention* OR barrier* OR enabler* OR facilitator* OR performance* OR improvement* OR optimizer* OR strateg* OR mechanism* OR productivit* OR tool* OR success* OR aceitação* OR hesitação OR rejeição OR recusa OR aceitabilidade OR intenção OR barreira* OR facilitador* OR melhoria* OR otimização OR estrategia* OR mecanismo* OR produtividade OR ferramenta* OR sucesso OR aceptación* OR vacilación OR rechazo OR aceptabilidad OR conocimiento OR barrera* OR mejora* OR optimización OR productividad OR herramienta* OR exito) AND (db:("LILACS")) AND (year_cluster:[2000 TO 2022])
**CINAHL**	(COVID-19 OR SARS-CoV-2 OR "Severe Acute Respiratory Syndrome Coronavirus 2" OR "Coronavirus Disease 2019" OR "2019 Novel Coronavirus" OR "2019 New Coronavirus" OR "Wuhan Coronavirus" OR 2019-nCoV OR HCoV-19 OR nCoV-2019 OR "Novel Coronavirus 2019-nCoV" OR "Alpha Variant" OR "Beta Variant" OR "Gamma Variant" OR "Delta Variant" OR "Delta Plus Variant" OR

	"Omicron Variant" OR "Lambda Variant") AND TI("Immunization Program" OR "Immunization Programs" OR "Mass Vaccination" OR "Mass Immunization" OR "Vaccination Programme" OR "Vaccination Programmes" OR "Vaccine Programme" OR "Vaccine Programmes" OR "Vaccination Team" OR "Vaccination Teams" OR "Vaccine Team" OR "Vaccine Teams" OR "Vaccination Campaign" OR "Vaccination Campaignes" OR "Vaccine Campaigne" OR "Vaccine Campaignes" OR Vaccination OR Immunization OR Antivaccine OR Anti-Vaccine OR Anti-Vaccination) AND TI(Refusal OR Acceptance* OR Hesitancy OR Acceptability OR Intention* OR Barrier* OR Enabler* OR Facilitator* OR Performance* OR Improvement* OR Optimizer* OR Strateg* OR Productivit* OR Mechanism* OR Productivity* OR Tool* OR Success*) AND (LA English OR LA Portuguese OR LA Spanish OR LA French OR LA Italian OR LA Hindi) AND (PY 2020 OR PY 2021 OR PY 2022)
**WHO COVID-19**	tw:((covid-19 OR sars-cov-2 OR "Severe Acute Respiratory Syndrome Coronavirus 2" OR "Coronavirus Disease 2019" OR "2019 Novel Coronavirus" OR "2019 New Coronavirus" OR "Wuhan Coronavirus" OR 2019-ncov OR hcov-19 OR ncov-2019 OR "Novel Coronavirus 2019-nCoV" OR "Alpha Variant" OR "Beta Variant" OR "Gama Variant" OR "Delta Variant" OR "Delta Plus Variant" OR "Omicron Variant" OR "Lambda Variant") AND ("Immunization Program" OR "Immunization Programs" OR "Mass Vaccination" OR "Mass Immunization" OR "Vaccination Programme" OR "Vaccination Programmes" OR "Vaccine Programme" OR "Vaccine Programmes" OR "Vaccination Team" OR "Vaccination Teams" OR "Vaccine Team" OR "Vaccine Teams" OR "Vaccination Campaign" OR "Vaccination Campaignes" OR "Vaccine Campaigne" OR "Vaccine Campaignes" OR Vaccination OR Immunization OR Antivaccine OR Anti-Vaccine OR Anti-Vaccination) AND (Refusal OR Acceptance* OR Hesitancy OR Acceptability OR Intention* OR Barrier* OR Enabler* OR Facilitator* OR Performance* OR Improvement* OR Optimizer* OR Strateg* OR Productivit* OR Mechanism* OR Productivity* OR Tool* OR Success*)) AND db:("GREY-COVIDWHO" OR "WHOIRIS") AND (year_cluster:[2020 TO 2022])
**GOOGLE**	allintitle: (covid-19 OR sars-cov-2 OR "Severe Acute Respiratory Syndrome Coronavirus 2" OR "Coronavirus Disease 2019" OR "2019 Novel Coronavirus" OR "2019 New Coronavirus" OR "Wuhan Coronavirus" OR 2019-ncov OR hcov-19 OR ncov-2019 OR "Novel

**SCHOLAR**	Coronavirus 2019-nCoV" OR "Alpha Variant" OR "Beta Variant" OR "Gama Variant" OR "Delta Variant" OR "Delta Plus Variant" OR "Omicron Variant" OR "Lambda Variant") AND (Vaccine* OR Vaccination* OR Immunization* OR Antivaccine* OR Anti-Vaccine* OR Anti-Vaccination*) AND (Refusal OR Acceptance* OR Hesitancy OR Acceptability OR Intention* OR Barrier* OR Enabler* OR Facilitator* OR Performance* OR Improvement* OR Optimizer* OR Strateg* OR Productivit* OR Mechanism* OR Productivit* OR Tool* OR Success*)

**DATABASE**	**SEARCH STRATEGIES – QUESTION 3**<br><br>Q3 “What are the barriers and mechanisms involved in improving vaccination teams’ performance and productivity?”
**PUBMED**	(((COVID-19[mh] OR SARS-CoV-2[mh] OR Severe Acute Respiratory Syndrome Coronavirus 2[tiab] OR Coronavirus Disease 2019[tiab] OR 2019 Novel Coronavirus[tiab] OR 2019 New Coronavirus[tiab] OR Wuhan Coronavirus[tiab] OR COVID-19[tiab] OR SARS-CoV-2[tiab] OR 2019-nCoV[tiab] OR HCoV-19[tiab] OR nCoV-2019[tiab] OR Novel Coronavirus 2019-nCoV[tiab] OR COVID-19 Vaccine*[tiab] OR COVID-19 Vaccination[tiab] OR Alpha Variant[tiab] OR Beta Variant[tiab] OR Gama Variant[tiab] OR Delta Variant[tiab] OR Delta Plus Variant[tiab] OR Omicron Variant[tiab] OR Lambda Variant[tiab]) AND (Health Workforce[mj] OR Workforce*[tiab] OR Health Manpower[tiab] OR Health Personnel[mj] OR Health Personnel*[tiab] OR Health Care Provider*[tiab] OR Healthcare Provider*[tiab] OR Health Care Worker*[tiab] OR Healthcare Worker*[tiab] OR Health Care Professional*[tiab] OR Healthcare Professional*[tiab] OR Human Resources for Health[tiab] OR Health Worker*[tiab] OR Allied Health Professional*[tiab] OR

	Healthcare Assistant*[tiab] OR Health Care Assistant*[tiab] OR Healthcare Support Worker*[tiab] OR Health Care Support Worker*[tiab] OR Caregivers[mj] OR Caregiver*[tiab] OR Licensed Practical Nurses[mj] OR Nursing Staff[mj] OR Nurses[mj] OR Nurse*[tiab] OR Nursing Personnel*[tiab] OR Nursing Staff*[tiab] OR Professional Nurse*[tiab] OR Nursing Associate*[tiab] OR Nursing Professional*[tiab] OR Nursing Assistant*[tiab] OR Auxiliary Nurse*[tiab] OR Nursing Auxiliar*[tiab] OR Licensed Practical Nurse*[tiab] OR Nursing Team*[tiab] OR Dentist*[tiab] OR Doctor*[tiab] OR Physicians[mj] OR Physician*[tiab] OR Pharmacist*[tiab] OR Physiotherapist*[tiab] OR Midwive*[tiab] OR Community Health Worker*[tiab] OR Community-Based Provider*[tiab] OR Laboratory Staff*[tiab] OR Paramedical Staff*[tiab] OR Paramedical Personnel*[tiab] OR Paramedic*[tiab])) AND (Requirement*[ti] OR Qualification*[ti] OR Precondition*[ti] OR Requisite*[ti] OR Prerequisite*[ti] OR Abilit*[ti] OR Skill*[ti])) NOT (Letter*[tw] OR Editorial*[tw] OR Release*[tw]) AND (English[lang] OR Portuguese[lang] OR Spanish[lang] OR French[lang] OR Italian[lang] OR Hindi[lang]) AND ("2020/01/01"[PDAT] : "2022/03/01"[PDAT])
**EMBASE**	('coronavirus disease 2019'/exp OR '2019 novel coronavirus disease':ti,ab OR '2019 novel coronavirus epidemic':ti,ab OR '2019 novel coronavirus infection':ti,ab OR '2019-ncov disease':ti,ab OR '2019-ncov infection':ti,ab OR 'COVID - 19':ti,ab OR 'COVID - 19 induced pneumonia':ti,ab OR 'covid 2019':ti,ab OR 'covid-19':ti,ab OR 'covid-19 induced pneumonia':ti,ab OR 'covid-19 pneumonia':ti,ab OR 'covid19':ti,ab OR 'sars coronavirus 2 infection':ti,ab OR 'sars coronavirus 2 pneumonia':ti,ab OR 'sars-cov-2 disease':ti,ab OR 'sars-cov-2 infection':ti,ab OR 'sars-cov-2 pneumonia':ti,ab OR 'sars-cov2 disease':ti,ab OR 'sars-cov2 infection':ti,ab OR 'sarscov2 disease':ti,ab OR 'sarscov2 infection':ti,ab OR 'wuhan coronavirus disease':ti,ab OR 'wuhan coronavirus infection':ti,ab OR 'coronavirus disease 2019':ti,ab OR 'coronavirus disease 2019 pneumonia':ti,ab OR 'coronavirus disease-19':ti,ab OR 'coronavirus infection 2019':ti,ab OR 'ncov 2019 disease':ti,ab OR 'ncov 2019 infection':ti,ab OR 'new coronavirus pneumonia':ti,ab OR 'novel coronavirus 2019 disease':ti,ab OR 'novel coronavirus 2019 infection':ti,ab OR 'novel coronavirus disease 2019':ti,ab OR 'novel coronavirus infected pneumonia':ti,ab OR 'novel coronavirus infection 2019':ti,ab OR 'novel coronavirus pneumonia':ti,ab OR 'paucisymptomatic coronavirus disease 2019':ti,ab OR

	'severe acute respiratory syndrome 2':ti,ab OR 'severe acute respiratory syndrome 2 pneumonia':ti,ab OR 'severe acute respiratory syndrome cov-2 infection':ti,ab OR 'severe acute respiratory syndrome coronavirus 2 infection':ti,ab OR 'severe acute respiratory syndrome coronavirus 2019 infection':ti,ab OR 'covid-19 vaccination':ti,ab OR 'covid-19 vaccine':ti,ab OR 'Alpha Variant':ti,ab OR 'Beta Variant':ti,ab OR 'Gama Variant':ti,ab OR 'Delta Variant':ti,ab OR 'Delta Plus Variant':ti,ab OR 'Omicron Variant':ti,ab OR 'Lambda Variant':ti,ab) AND ('health workforce'/mj OR 'health care labour force':ti,ab OR 'health care manpower':ti,ab OR 'health care work force':ti,ab OR 'health care workforce':ti,ab OR 'health labor force':ti,ab OR 'health labour force':ti,ab OR 'health manpower':ti,ab OR 'health work force':ti,ab OR 'health workforce':ti,ab OR 'healthcare labor force':ti,ab OR 'healthcare labour force':ti,ab OR 'healthcare manpower':ti,ab OR 'healthcare work force':ti,ab OR 'healthcare workforce':ti,ab OR 'health care labor force':ti,ab OR 'health care personnel'/mj OR 'health care personnel*':ti,ab OR 'health care practitioner*':ti,ab OR 'health care professional*':ti,ab OR 'health care provider*':ti,ab OR 'health care worker*':ti,ab OR 'health personnel*':ti,ab OR 'health worker*':ti,ab OR 'healthcare personnel':ti,ab OR 'healthcare practitioner':ti,ab OR 'healthcare professional':ti,ab OR 'healthcare provider':ti,ab OR 'healthcare worker*':ti,ab OR 'caregiver'/mj OR 'caregiver*':ti,ab OR 'human resources for health':ti,ab OR 'licensed practical nurse'/mj OR 'licensed practical nurse*':ti,ab OR 'licensed vocational nurse':ti,ab OR 'nursing staff'/mj OR 'hospital nursing staff':ti,ab OR 'nurse staffing':ti,ab OR 'nursing manpower':ti,ab OR 'nursing personnel':ti,ab OR 'nursing staff':ti,ab OR 'nurse'/mj OR 'nurse*':ti,ab OR 'nursing associate*':ti,ab OR 'nursing assistant'/mj OR 'nursing assistant*':ti,ab OR 'auxiliary nurse*':ti,ab OR 'nursing auxiliar*':ti,ab OR 'team nursing'/mj OR 'team nursing':ti,ab OR 'dentist'/mj OR 'dentist*':ti,ab OR 'physician'/mj OR 'doctor*':ti,ab OR 'physician*':ti,ab OR 'private physician':ti,ab OR 'pharmacist'/mj OR 'pharmacist*':ti,ab OR 'physiotherapist'/mj OR 'physical therapist*':ti,ab OR 'physiotherapist*':ti,ab OR 'midwife'/mj OR 'midwife':ti,ab OR 'midwifery':ti,ab OR 'midwives':ti,ab OR 'health auxiliary'/mj OR 'auxiliary health worker':ti,ab OR 'community health worker*':ti,ab OR 'medical auxiliary':ti,ab OR 'community-based provider*':ti,ab OR 'laboratory staff*':ti,ab OR 'paramedical personnel'/mj OR 'healthcare assistant':ti,ab OR 'healthcare support worker':ti,ab OR 'para

	medical personnel':ti,ab OR 'paramedical personnel':ti,ab OR 'paramedical professional':ti,ab OR 'paramedical staff':ti,ab) AND (requirement*:ti OR qualification*:ti OR precondition*:ti OR requisite*:ti OR prerequisite*:ti OR abilit*:ti OR skill*:ti) AND ([english]/lim OR [french]/lim OR [hindi]/lim OR [italian]/lim OR [portuguese]/lim OR [spanish]/lim) AND [01-01-2020]/sd NOT [01-03-2022]/sd AND [embase]/lim NOT ([embase]/lim AND [medline]/lim)
**SCOPUS**	ALL(COVID-19 OR SARS-CoV-2 OR "Severe Acute Respiratory Syndrome Coronavirus 2" OR "Coronavirus Disease 2019" OR "2019 Novel Coronavirus" OR "2019 New Coronavirus" OR "Wuhan Coronavirus" OR 2019-nCoV OR HCoV-19 OR nCoV-2019 OR "Novel Coronavirus 2019-nCoV" OR "Alpha Variant" OR "Beta Variant" OR "Gama Variant" OR "Delta Variant" OR "Delta Plus Variant" OR "Omicron Variant" OR "Lambda Variant") AND TITLE("Health Workforce" OR "Health Workforces" OR "Health Manpower" OR "Health Personnel" OR "Health Personnels" OR "Health Care Providers" OR "Healthcare Providers" OR "Health Care Workers" OR "Healthcare Workers" OR "Health Care Professionals" OR "Healthcare Professionals" OR Caregiver* OR "Licensed Practical Nurses" OR "Nursing Staff" OR Nurse* OR "Nursing Personnel" OR "Professional Nurses" OR "Nursing Associate" OR "Nursing Professionals" OR "Nursing Assistant" OR "Auxiliary Nurses" OR "Nursing Auxiliary" OR "Licensed Practical Nurses" OR "Nursing Team" OR Dentists OR Doctors OR Physicians OR Pharmacists OR Physiotherapists OR Midwives OR "Community Health Workers" OR "Community-Based Providers" OR "Laboratory Staff" OR "Paramedical Staff" OR "Paramedical Personnel") AND TITLE(Requirement* OR Qualification* OR Precondition* OR Requisite* OR Prerequisite* OR Abilit* OR Skill*) AND (LIMIT-TO(DOCTYPE, "ar")) AND (LIMIT-TO(LANGUAGE, "English") OR LIMIT-TO(LANGUAGE, "Spanish") OR LIMIT-TO(LANGUAGE, "French") OR LIMIT-TO(LANGUAGE, "Portuguese")) OR LIMIT-TO(LANGUAGE, "Italian")) OR LIMIT-TO(LANGUAGE, "Hindi")) AND (LIMIT-TO(PUBYEAR, 2022) OR LIMIT-TO(PUBYEAR, 2021) OR LIMIT-TO(PUBYEAR, 2020))

**BVS/LILACS**	(covid-19 OR sars-cov-2 OR "Severe Acute Respiratory Syndrome Coronavirus 2" OR "Coronavirus Disease 2019" OR "2019 Novel Coronavirus" OR "2019 New Coronavirus" OR "Wuhan Coronavirus" OR 2019-ncov OR hcov-19 OR ncov-2019 OR "Novel Coronavirus 2019-nCoV" OR "COVID-19 Vaccines" OR "COVID-19 Vaccination" OR "Alpha Variant" OR "Beta Variant" OR "Gama Variant" OR "Delta Variant" OR "Delta Plus Variant" OR "Omicron Variant" OR "Lambda Variant") AND ("Health Workforce" OR "Health Workforces" OR "Health Manpower" OR "Health Personnel" OR "Health Personnels" OR "Health Care Providers" OR "Healthcare Providers" OR "Health Care Workers" OR "Healthcare Workers" OR "Health Care Professionals" OR "Healthcare Professionals" OR caregiver* OR "Licensed Practical Nurses" OR "Nursing Staff" OR Nurse* OR "Nursing Personnel" OR "Professional Nurses" OR "Nursing Associate" OR "Nursing Professionals" OR "Nursing Assistant" OR "Auxiliary Nurses" OR "Nursing Auxiliary" OR "Licensed Practical Nurses" OR "Nursing Team" OR Dentist* OR Doctor* OR Physicians OR Pharmacist* OR Physiotherapist* OR Midwive* OR "Community Health Workers" OR "Community-Based Providers" OR "Laboratory Staff" OR Paramedical OR "força de trabalho em saúde" OR "recursos humanos em saúde" OR "pessoal de saúde" OR "provedores de saúde" OR "trabalhadores em saúde" OR "profissionais de saúde" OR cuidador* OR enfermeir* OR "equipe de enfermagem" OR "pessoal de enfermagem" OR "profissionais de enfermagem" OR "assistente de enfermagem" OR "auxiliares de enfermagem" OR dentista* OR médico* OR farmacêutico* OR fisioterapeuta* OR parteira* OR "trabalhadores comunitários de saúde" OR "provedores de base comunitária" OR "equipe de laboratório" OR paramédico* OR "personal de salud" OR "recursos humanos en salud" OR "proveedores de salud" OR "trabajadores de la salud" OR "servicios profesionales de salud" OR "personal de enfermería" OR enfermera* OR "personal de enfermería" OR "profesionales de enfermería" OR "auxiliar de enfermería" OR "auxiliares de enfermería" OR partera* OR "trabajadores de salud comunitarios" OR "proveedores comunitarios" OR "personal de laboratorio") AND (ti:(requirement* OR qualification* OR precondition* OR requisite* OR prerequisite* OR abilit* OR skill*)) AND (db:("LILACS"))

**CINAHL**	(COVID-19 OR SARS-CoV-2 OR "Severe Acute Respiratory Syndrome Coronavirus 2" OR "Coronavirus Disease 2019" OR "2019 Novel Coronavirus" OR "2019 New Coronavirus" OR "Wuhan Coronavirus" OR 2019-nCoV OR HCoV-19 OR nCoV-2019 OR "Novel Coronavirus 2019-nCoV" OR "COVID-19 Vaccines" OR "COVID-19 Vaccination" OR "Alpha Variant" OR "Beta Variant" OR "Gama Variant" OR "Delta Variant" OR "Delta Plus Variant" OR "Omicron Variant" OR "Lambda Variant") AND ("Health Workforce" OR "Health Workforces" OR "Health Manpower" OR "Health Personnel" OR "Health Personnels" OR "Health Care Providers" OR "Healthcare Providers" OR "Health Care Workers" OR "Healthcare Workers" OR "Health Care Professionals" OR "Healthcare Professionals" OR Caregiver* OR "Licensed Practical Nurses" OR "Nursing Staff" OR Nurse* OR "Nursing Personnel" OR "Professional Nurses" OR "Nursing Associate" OR "Nursing Professionals" OR "Nursing Assistant" OR "Auxiliary Nurses" OR "Nursing Auxiliary" OR "Licensed Practical Nurses" OR "Nursing Team" OR Dentists OR Doctors OR Physicians OR Pharmacists OR Physiotherapists OR Midwives OR "Community Health Workers" OR "Community-Based Providers" OR "Laboratory Staff" OR "Paramedical Staff" OR "Paramedical Personnel") AND TI(Requirement* OR Qualification* OR Precondition* OR Requisite* OR Prerequisite* OR Abilit* OR Skill*) AND (LA English OR LA Portuguese OR LA Spanish OR LA French OR LA Italian OR LA Hindi) AND (PY 2020 OR PY 2021 OR PY 2022)
**WHO COVID-19**	(COVID-19 OR SARS-CoV-2 OR "Severe Acute Respiratory Syndrome Coronavirus 2" OR "Coronavirus Disease 2019" OR "2019 Novel Coronavirus" OR "2019 New Coronavirus" OR "Wuhan Coronavirus" OR 2019-nCoV OR HCoV-19 OR nCoV-2019 OR "Novel Coronavirus 2019-nCoV" OR "COVID-19 Vaccines" OR "COVID-19 Vaccination" OR "Alpha Variant" OR "Beta Variant" OR "Gama Variant" OR "Delta Variant" OR "Delta Plus Variant" OR "Omicron Variant" OR "Lambda Variant") AND ("Health Workforce" OR "Health Workforces" OR "Health Manpower" OR "Health Personnel" OR "Health Personnels" OR "Health Care Providers" OR "Healthcare Providers" OR "Health Care Workers" OR "Healthcare Workers" OR "Health Care Professionals" OR "Healthcare Professionals" OR Caregiver* OR "Licensed Practical Nurses" OR "Nursing Staff" OR Nurse* OR "Nursing Personnel" OR "Professional

	Nurses" OR "Nursing Associate" OR "Nursing Professionals" OR "Nursing Assistant" OR "Auxiliary Nurses" OR "Nursing Auxiliary" OR "Licensed Practical Nurses" OR "Nursing Team" OR Dentists OR Doctors OR Physicians OR Pharmacists OR Physiotherapists OR Midwives OR "Community Health Workers" OR "Community-Based Providers" OR "Laboratory Staff" OR "Paramedical Staff" OR "Paramedical Personnel") AND (Requirement* OR Qualification* OR Precondition* OR Requisite* OR Prerequisite* OR Abilit* OR Skill*) AND db:("GREY-COVIDWHO" OR "WHOIRIS")
**GOOGLE SCHOLAR**	allintitle: (COVID-19 OR SARS-CoV-2 OR "Severe Acute Respiratory Syndrome Coronavirus 2" OR "Coronavirus Disease 2019" OR "2019 Novel Coronavirus" OR "2019 New Coronavirus" OR "Wuhan Coronavirus" OR 2019-nCoV OR HCoV-19 OR nCoV-2019 OR "Novel Coronavirus 2019-nCoV" OR "COVID-19 Vaccines" OR "COVID-19 Vaccination" OR "Alpha Variant" OR "Beta Variant" OR "Gama Variant" OR "Delta Variant" OR "Delta Plus Variant" OR "Omicron Variant" OR "Lambda Variant") AND ("Health Workforce" OR "Health Workforces" OR "Health Manpower" OR "Health Personnel" OR "Health Personnels" OR "Health Care Providers" OR "Healthcare Providers" OR "Health Care Workers" OR "Healthcare Workers" OR "Health Care Professionals" OR "Healthcare Professionals" OR Caregiver* OR "Licensed Practical Nurses" OR "Nursing Staff" OR Nurse* OR "Nursing Personnel" OR "Professional Nurses" OR "Nursing Associate" OR "Nursing Professionals" OR "Nursing Assistant" OR "Auxiliary Nurses" OR "Nursing Auxiliary" OR "Licensed Practical Nurses" OR "Nursing Team" OR Dentists OR Doctors OR Physicians OR Pharmacists OR Physiotherapists OR Midwives OR "Community Health Workers" OR "Community-Based Providers" OR "Laboratory Staff" OR "Paramedical Staff" OR "Paramedical Personnel") AND (Requirement* OR Qualification* OR Precondition* OR Requisite* OR Prerequisite* OR Abilit* OR Skill*)

DATABASE	**SEARCH STRATEGIES – QUESTION 4**<br><br>Q4 "What are the main barriers and enablers for countries to obtain the desired or required COVID-19 vaccination coverage for HCW and the target population?"
**PUBMED**	((((COVID-19[mh] OR SARS-CoV-2[mh] OR Severe Acute Respiratory Syndrome Coronavirus 2[tiab] OR Coronavirus Disease 2019[tiab] OR 2019 Novel Coronavirus[tiab] OR 2019 New Coronavirus[tiab] OR Wuhan Coronavirus[tiab] OR COVID-19[tiab] OR SARS-CoV-2[tiab] OR 2019-nCoV[tiab] OR HCoV-19[tiab] OR nCoV-2019[tiab] OR Novel Coronavirus 2019-nCoV[tiab] OR Alpha Variant[tiab] OR Beta Variant[tiab] OR Gama Variant[tiab] OR Delta Variant[tiab] OR Delta Plus Variant[tiab] OR Omicron Variant[tiab] OR Lambda Variant[tiab) AND (COVID-19 Vaccines[mh] OR Vaccination[mj] OR Vaccination Coverage[mj] OR Vaccination Coverage[tiab] OR Vaccination[tiab] OR Immunization[tiab] OR Vaccine*[tiab])) AND (Health Workforce[mj] OR Workforce*[tiab] OR Health Manpower[tiab] OR Health Personnel[mj] OR Health Personnel*[tiab] OR Health Care Provider*[tiab] OR Healthcare Provider*[tiab] OR Health Care Worker*[tiab] OR Healthcare Worker*[tiab] OR Health Care Professional*[tiab] OR Healthcare Professional*[tiab] OR Human Resources for Health[tiab] OR Health Worker*[tiab] OR Allied Health Professional*[tiab] OR Healthcare Assistant*[tiab] OR Health Care Assistant*[tiab] OR Healthcare Support Worker*[tiab] OR Health Care Support Worker*[tiab] OR Caregivers[mj] OR Caregiver*[tiab] OR Licensed Practical Nurses[mj] OR Nursing Staff[mj] OR Nurses[mj] OR Nurse*[tiab] OR Nursing Personnel*[tiab] OR Nursing Staff*[tiab] OR Professional Nurse*[tiab] OR Nursing Associate*[tiab] OR Nursing Professional*[tiab] OR Nursing Assistant*[tiab] OR Auxiliary Nurse*[tiab] OR Nursing Auxiliar*[tiab] OR Licensed Practical Nurse*[tiab] OR Nursing Team*[tiab] OR Dentist*[tiab] OR Doctor*[tiab] OR Physicians[mj] OR Physician*[tiab] OR Pharmacist*[tiab] OR Physiotherapist*[tiab] OR Midwive*[tiab] OR Community Health Worker*[tiab] OR Community-Based Provider*[tiab] OR Laboratory Staff*[tiab] OR

	Paramedical Staff*[tiab] OR Paramedical Personnel*[tiab] OR Paramedic*[tiab] OR Health Facilit*[tiab] OR Hospital*[ti] OR Health Institution*[tiab] OR Health*[ti])) AND (Mandatory Programs[mh] OR Policy[mh] OR Polic*[ti] OR Political*[ti] OR Regulation*[ti] OR Prioritization*[ti] OR Prioritize*[ti] OR Prioritise*[ti] OR Mandat*[ti] OR Law[ti] OR Laws[ti] OR Right*[ti] OR Rule*[ti] OR Obligatoriness[ti] OR Regulatory[ti])) NOT (Letter*[tw] OR Editorial*[tw] OR Release*[tw]) AND (English[lang] OR Portuguese[lang] OR Spanish[lang] OR French[lang] OR Italian[lang] OR Hindi[lang]) AND ("2020/01/01"[PDAT] : "2022/03/01"[PDAT])
**EMBASE**	('coronavirus disease 2019'/exp OR '2019 novel coronavirus disease':ti,ab OR '2019 novel coronavirus epidemic':ti,ab OR '2019 novel coronavirus infection':ti,ab OR '2019-ncov disease':ti,ab OR '2019-ncov infection':ti,ab OR 'COVID - 19':ti,ab OR 'COVID - 19 induced pneumonia':ti,ab OR 'covid 2019':ti,ab OR 'covid-19':ti,ab OR 'covid-19 induced pneumonia':ti,ab OR 'covid-19 pneumonia':ti,ab OR 'covid19':ti,ab OR 'sars coronavirus 2 infection':ti,ab OR 'sars coronavirus 2 pneumonia':ti,ab OR 'sars-cov-2 disease':ti,ab OR 'sars-cov-2 infection':ti,ab OR 'sars-cov-2 pneumonia':ti,ab OR 'sars-cov2 disease':ti,ab OR 'sars-cov2 infection':ti,ab OR 'sarscov2 disease':ti,ab OR 'sarscov2 infection':ti,ab OR 'wuhan coronavirus disease':ti,ab OR 'wuhan coronavirus infection':ti,ab OR 'coronavirus disease 2019':ti,ab OR 'coronavirus disease 2019 pneumonia':ti,ab OR 'coronavirus disease-19':ti,ab OR 'coronavirus infection 2019':ti,ab OR 'ncov 2019 disease':ti,ab OR 'ncov 2019 infection':ti,ab OR 'new coronavirus pneumonia':ti,ab OR 'novel coronavirus 2019 disease':ti,ab OR 'novel coronavirus 2019 infection':ti,ab OR 'novel coronavirus disease 2019':ti,ab OR 'novel coronavirus infected pneumonia':ti,ab OR 'novel coronavirus infection 2019':ti,ab OR 'novel coronavirus pneumonia':ti,ab OR 'paucisymptomatic coronavirus disease 2019':ti,ab OR 'severe acute respiratory syndrome 2':ti,ab OR 'severe acute respiratory syndrome 2 pneumonia':ti,ab OR 'severe acute respiratory syndrome cov-2 infection':ti,ab OR 'severe acute respiratory syndrome coronavirus 2 infection':ti,ab OR 'severe acute respiratory syndrome coronavirus 2019 infection':ti,ab OR 'Alpha Variant':ti,ab OR 'Beta Variant':ti,ab OR 'Gama Variant':ti,ab OR 'Delta Variant':ti,ab OR 'Delta Plus Variant':ti,ab OR 'Omicron Variant':ti,ab OR 'Lambda Variant':ti,ab) AND ('immunization program*':ti,ab OR 'mass immunization'/exp OR 'mass immunisation' OR 'mass immunization' OR 'mass vaccination' OR 'vaccination'/exp OR

	'vaccination program':ti,ab OR 'vaccination programme':ti,ab OR 'vaccine programm*':ti,ab OR 'vaccination team*':ti,ab OR 'vaccine team*':ti,ab OR 'vaccination campaign*':ti,ab OR 'vaccine campaign*':ti,ab OR vaccination*:ti) AND ('health workforce'/mj OR 'health care labour force':ti,ab OR 'health care manpower':ti,ab OR 'health care work force':ti,ab OR 'health care workforce':ti,ab OR 'health labor force':ti,ab OR 'health labour force':ti,ab OR 'health manpower':ti,ab OR 'health work force':ti,ab OR 'health workforce':ti,ab OR 'healthcare labor force':ti,ab OR 'healthcare labour force':ti,ab OR 'healthcare manpower':ti,ab OR 'healthcare work force':ti,ab OR 'healthcare workforce':ti,ab OR 'health care labor force':ti,ab OR 'health care personnel'/mj OR 'health care personnel*':ti,ab OR 'health care practitioner*':ti,ab OR 'health care professional*':ti,ab OR 'health care provider*':ti,ab OR 'health care worker*':ti,ab OR 'health personnel*':ti,ab OR 'health worker*':ti,ab OR 'healthcare personnel':ti,ab OR 'healthcare practitioner':ti,ab OR 'healthcare professional':ti,ab OR 'healthcare provider':ti,ab OR 'healthcare worker*':ti,ab OR 'caregiver'/mj OR 'caregiver*':ti,ab OR 'human resources for health':ti,ab OR 'licensed practical nurse'/mj OR 'licensed practical nurse*':ti,ab OR 'licensed vocational nurse':ti,ab OR 'nursing staff'/mj OR 'hospital nursing staff':ti,ab OR 'nurse staffing':ti,ab OR 'nursing manpower':ti,ab OR 'nursing personnel':ti,ab OR 'nursing staff':ti,ab OR 'nurse'/mj OR 'nurse*':ti,ab OR 'nursing associate*':ti,ab OR 'nursing assistant'/mj OR 'nursing assistant*':ti,ab OR 'auxiliary nurse*':ti,ab OR 'nursing auxiliar*':ti,ab OR 'team nursing'/mj OR 'team nursing':ti,ab OR 'dentist'/mj OR 'dentist*':ti,ab OR 'physician'/mj OR 'doctor*':ti,ab OR 'physician*':ti,ab OR 'private physician':ti,ab OR 'pharmacist'/mj OR 'pharmacist*':ti,ab OR 'physiotherapist'/mj OR 'physical therapist*':ti,ab OR 'physiotherapist*':ti,ab OR 'midwife'/mj OR 'midwife':ti,ab OR 'midwifery':ti,ab OR 'midwives':ti,ab OR 'health auxiliary'/mj OR 'auxiliary health worker':ti,ab OR 'community health worker*':ti,ab OR 'medical auxiliary':ti,ab OR 'community-based provider*':ti,ab OR 'laboratory staff*':ti,ab OR 'paramedical personnel'/mj OR 'healthcare assistant':ti,ab OR 'healthcare support worker':ti,ab OR 'para medical personnel':ti,ab OR 'paramedical personnel':ti,ab OR 'paramedical professional':ti,ab OR 'paramedical staff':ti,ab) AND ('mandatory program'/mj OR 'mandatory program*':ti OR 'policy'/exp OR 'policy':ti OR political*:ti OR regulation*:ti OR prioritization*:ti OR prioritize*:ti OR prioritise*:ti OR mandat*:ti OR 'law':ti OR right*:ti OR rule*:ti

	OR obligatoriness:ti OR regulatory:ti) AND ([english]/lim OR [french]/lim OR [hindi]/lim OR [italian]/lim OR [portuguese]/lim OR [spanish]/lim) AND [01-01-2020]/sd NOT [03-01-2022]/sd AND [embase]/lim NOT ([embase]/lim AND [medline]/lim)
**SCOPUS**	ALL(COVID-19 OR SARS-CoV-2 OR "Severe Acute Respiratory Syndrome Coronavirus 2" OR "Coronavirus Disease 2019" OR "2019 Novel Coronavirus" OR "2019 New Coronavirus" OR "Wuhan Coronavirus" OR 2019-nCoV OR HCoV-19 OR nCoV-2019 OR "Novel Coronavirus 2019-nCoV" OR "Alpha Variant" OR "Beta Variant" OR "Gama Variant" OR "Delta Variant" OR "Delta Plus Variant" OR "Omicron Variant" OR "Lambda Variant") AND ALL("Immunization Program" OR "Immunization Programs" OR "Mass Vaccination" OR "Mass Immunization" OR "Vaccination Programme" OR "Vaccination Programmes" OR "Vaccine Programme" OR "Vaccine Programmes" OR "Vaccination Team" OR "Vaccination Teams" OR "Vaccine Team" OR "Vaccine Teams" OR "Vaccination Campaign" OR "Vaccination Campaignes" OR "Vaccine Campaigne" OR "Vaccine Campaignes" OR Vaccination OR Immunization) AND ALL("Health Workforce" OR "Health Workforces" OR "Health Manpower" OR "Health Personnel" OR "Health Personnels" OR "Health Care Providers" OR "Healthcare Providers" OR "Health Care Workers" OR "Healthcare Workers" OR "Health Care Professionals" OR "Healthcare Professionals" OR Caregiver* OR "Licensed Practical Nurses" OR "Nursing Staff" OR Nurse* OR "Nursing Personnel" OR "Professional Nurses" OR "Nursing Associate" OR "Nursing Professionals" OR "Nursing Assistant" OR "Auxiliary Nurses" OR "Nursing Auxiliary" OR "Licensed Practical Nurses" OR "Nursing Team" OR Dentists OR Doctors OR Physicians OR Pharmacists OR Physiotherapists OR Midwives OR "Community Health Workers" OR "Community-Based Providers" OR "Laboratory Staff" OR "Paramedical Staff" OR "Paramedical Personnel") AND TITLE("Mandatory Programs" OR Polic* OR Political* OR Regulation* OR Prioritization* OR Prioritize* OR Prioritise* OR Mandat* OR Law OR Laws OR Right* OR Rule* OR Obligatoriness OR Regulatory) AND (LIMIT-TO(DOCTYPE, "ar")) AND (LIMIT-TO(LANGUAGE, "English") OR LIMIT-TO(LANGUAGE, "Spanish") OR LIMIT-TO(LANGUAGE, "French") OR LIMIT-TO(LANGUAGE, "Portuguese")) OR LIMIT-TO(LANGUAGE, "Italian")) OR LIMIT-TO(LANGUAGE, "Hindi")) AND (LIMIT-TO(PUBYEAR, 2022) OR LIMIT-TO(PUBYEAR, 2021) OR LIMIT-TO(PUBYEAR, 2020))

**BVS/LILACS**	(covid-19 OR sars-cov-2 OR "Severe Acute Respiratory Syndrome Coronavirus 2" OR "Coronavirus Disease 2019" OR "2019 Novel Coronavirus" OR "2019 New Coronavirus" OR "Wuhan Coronavirus" OR 2019-ncov OR hcov-19 OR ncov-2019 OR "Novel Coronavirus 2019-nCoV" OR "Alpha Variant" OR "Beta Variant" OR "Gama Variant" OR "Delta Variant" OR "Delta Plus Variant" OR "Omicron Variant" OR "Lambda Variant") AND ("COVID-19 Vaccines" OR vaccination OR "Vaccination Coverage" OR "Vaccination Coverage" OR vaccination OR immunization OR vaccine* OR vacina* OR anti-vacina OR imunização OR vacuna* OR inmunización) AND ("Health Workforce" OR "Health Workforces" OR "Health Manpower" OR "Health Personnel" OR "Health Personnels" OR "Health Care Providers" OR "Healthcare Providers" OR "Health Care Workers" OR "Healthcare Workers" OR "Health Care Professionals" OR "Healthcare Professionals" OR caregiver* OR "Licensed Practical Nurses" OR "Nursing Staff" OR Nurse* OR "Nursing Personnel" OR "Professional Nurses" OR "Nursing Associate" OR "Nursing Professionals" OR "Nursing Assistant" OR "Auxiliary Nurses" OR "Nursing Auxiliary" OR "Licensed Practical Nurses" OR "Nursing Team" OR Dentist* OR Doctor* OR Physicians OR Pharmacist* OR Physiotherapist* OR Midwive* OR "Community Health Workers" OR "Community-Based Providers" OR "Laboratory Staff" OR Paramedical OR "força de trabalho em saúde" OR "recursos humanos em saúde" OR "pessoal de saúde" OR "provedores de saúde" OR "trabalhadores em saúde" OR "profissionais de saúde" OR cuidador* OR enfermeir* OR "equipe de enfermagem" OR "pessoal de enfermagem" OR "profissionais de enfermagem" OR "assistente de enfermagem" OR "auxiliares de enfermagem" OR dentista* OR médico* OR farmacêutico* OR fisioterapeuta* OR parteira* OR "trabalhadores comunitários de saúde" OR "provedores de base comunitária" OR "equipe de laboratório" OR paramédico* OR "personal de salud" OR "recursos humanos en salud" OR "proveedores de salud" OR "trabajadores de la salud" OR "servicios profesionales de salud" OR "personal de enfermería" OR enfermera* OR "personal de enfermería" OR "profesionales de enfermería" OR "auxiliar de enfermería" OR "auxiliares de enfermería" OR partera* OR "trabajadores de salud comunitarios" OR "proveedores comunitarios" OR "personal de laboratorio") AND ("Mandatory Programs" OR Polic* OR Political* OR Regulation* OR Prioritization* OR Prioritize* OR Prioritise* OR Mandat* OR Law OR Laws OR Right* OR Rule*

	OR Obligatoriness OR Regulatory OR Compulsory OR Política* OR Regulamentac* OR Priorizac* OR Priorizar* OR Lei OR Leis OR Direito* OR Regra* OR Obrigac OR Regulac* OR Obrigatoriedade OR Regulación* OR Ley OR Leyes OR Regla* OR Obligación OR Obligatori*) AND (db:("LILACS"))
**CINAHL**	(COVID-19 OR SARS-CoV-2 OR "Severe Acute Respiratory Syndrome Coronavirus 2" OR "Coronavirus Disease 2019" OR "2019 Novel Coronavirus" OR "2019 New Coronavirus" OR "Wuhan Coronavirus" OR 2019-nCoV OR HCoV-19 OR nCoV-2019 OR "Novel Coronavirus 2019-nCoV" OR "Alpha Variant" OR "Beta Variant" OR "Gama Variant" OR "Delta Variant" OR "Delta Plus Variant" OR "Omicron Variant" OR "Lambda Variant") AND ("Immunization Program" OR "Immunization Programs" OR "Mass Vaccination" OR "Mass Immunization" OR "Vaccination Programme" OR "Vaccination Programmes" OR "Vaccine Programme" OR "Vaccine Programmes" OR "Vaccination Team" OR "Vaccination Teams" OR "Vaccine Team" OR "Vaccine Teams" OR "Vaccination Campaign" OR "Vaccination Campaignes" OR "Vaccine Campaigne" OR "Vaccine Campaignes" OR Vaccination OR Immunization) AND ("Health Workforce" OR "Health Workforces" OR "Health Manpower" OR "Health Personnel" OR "Health Personnels" OR "Health Care Providers" OR "Healthcare Providers" OR "Health Care Workers" OR "Healthcare Workers" OR "Health Care Professionals" OR "Healthcare Professionals" OR Caregiver* OR "Licensed Practical Nurses" OR "Nursing Staff" OR Nurse* OR "Nursing Personnel" OR "Professional Nurses" OR "Nursing Associate" OR "Nursing Professionals" OR "Nursing Assistant" OR "Auxiliary Nurses" OR "Nursing Auxiliary" OR "Licensed Practical Nurses" OR "Nursing Team" OR Dentists OR Doctors OR Physicians OR Pharmacists OR Physiotherapists OR Midwives OR "Community Health Workers" OR "Community-Based Providers" OR "Laboratory Staff" OR "Paramedical Staff" OR "Paramedical Personnel") AND TI("Mandatory Programs" OR Polic* OR Political* OR Regulation* OR Prioritization* OR Prioritize* OR Prioritise* OR Mandat* OR Law OR Laws OR Right* OR Rule* OR Obligatoriness OR Regulatory OR Compulsory)

**WHO COVID-19**	(COVID-19 OR SARS-CoV-2 OR "Severe Acute Respiratory Syndrome Coronavirus 2" OR "Coronavirus Disease 2019" OR "2019 Novel Coronavirus" OR "2019 New Coronavirus" OR "Wuhan Coronavirus" OR 2019-nCoV OR HCoV-19 OR nCoV-2019 OR "Novel Coronavirus 2019-nCoV" OR "Alpha Variant" OR "Beta Variant" OR "Gama Variant" OR "Delta Variant" OR "Delta Plus Variant" OR "Omicron Variant" OR "Lambda Variant") AND ("Immunization Program" OR "Immunization Programs" OR "Mass Vaccination" OR "Mass Immunization" OR "Vaccination Programme" OR "Vaccination Programmes" OR "Vaccine Programme" OR "Vaccine Programmes" OR "Vaccination Team" OR "Vaccination Teams" OR "Vaccine Team" OR "Vaccine Teams" OR "Vaccination Campaign" OR "Vaccination Campaignes" OR "Vaccine Campaigne" OR "Vaccine Campaignes" OR Vaccination OR Immunization) AND ("Health Workforce" OR "Health Workforces" OR "Health Manpower" OR "Health Personnel" OR "Health Personnels" OR "Health Care Providers" OR "Healthcare Providers" OR "Health Care Workers" OR "Healthcare Workers" OR "Health Care Professionals" OR "Healthcare Professionals" OR Caregiver* OR "Licensed Practical Nurses" OR "Nursing Staff" OR Nurse* OR "Nursing Personnel" OR "Professional Nurses" OR "Nursing Associate" OR "Nursing Professionals" OR "Nursing Assistant" OR "Auxiliary Nurses" OR "Nursing Auxiliary" OR "Licensed Practical Nurses" OR "Nursing Team" OR Dentists OR Doctors OR Physicians OR Pharmacists OR Physiotherapists OR Midwives OR "Community Health Workers" OR "Community-Based Providers" OR "Laboratory Staff" OR "Paramedical Staff" OR "Paramedical Personnel") AND ("Mandatory Programs" OR Polic* OR Political* OR Regulation* OR Prioritization* OR Prioritize* OR Prioritise* OR Mandat* OR Law OR Laws OR Right* OR Rule* OR Obligatoriness OR Regulatory OR Compulsory) AND db:("GREY-COVIDWHO" OR "WHOIRIS")
**GOOGLE SCHOLAR**	allintitle: (COVID-19 OR SARS-CoV-2 OR "Severe Acute Respiratory Syndrome Coronavirus 2" OR "Coronavirus Disease 2019" OR "2019 Novel Coronavirus" OR "2019 New Coronavirus" OR "Wuhan Coronavirus" OR 2019-nCoV OR HCoV-19 OR nCoV-2019 OR "Novel Coronavirus 2019-nCoV" OR "Alpha Variant" OR "Beta Variant" OR "Gama Variant" OR "Delta Variant" OR "Delta Plus Variant" OR "Omicron Variant" OR "Lambda Variant") AND ("Immunization Program" OR "Immunization Programs" OR "Mass Vaccination"

	OR "Mass Immunization" OR "Vaccination Programme" OR "Vaccination Programmes" OR "Vaccine Programme" OR "Vaccine Programmes" OR "Vaccination Team" OR "Vaccination Teams" OR "Vaccine Team" OR "Vaccine Teams" OR "Vaccination Campaign" OR "Vaccination Campaignes" OR "Vaccine Campaigne" OR "Vaccine Campaignes" OR Vaccination OR Immunization) AND ("Health Workforce" OR "Health Workforces" OR "Health Manpower" OR "Health Personnel" OR "Health Personnels" OR "Health Care Providers" OR "Healthcare Providers" OR "Health Care Workers" OR "Healthcare Workers" OR "Health Care Professionals" OR "Healthcare Professionals" OR Caregiver* OR "Licensed Practical Nurses" OR "Nursing Staff" OR Nurse* OR "Nursing Personnel" OR "Professional Nurses" OR "Nursing Associate" OR "Nursing Professionals" OR "Nursing Assistant" OR "Auxiliary Nurses" OR "Nursing Auxiliary" OR "Licensed Practical Nurses" OR "Nursing Team" OR Dentists OR Doctors OR Physicians OR Pharmacists OR Physiotherapists OR Midwives OR "Community Health Workers" OR "Community-Based Providers" OR "Laboratory Staff" OR "Paramedical Staff" OR "Paramedical Personnel") AND ("Mandatory Programs" OR Polic* OR Political* OR Regulation* OR Prioritization* OR Prioritize* OR Prioritise* OR Mandat* OR Law OR Laws OR Right* OR Rule* OR Obligatoriness OR Regulatory OR Compulsory)

Annex S3. Data extraction

Record ID	
Title	
Author	
Publication Year	( ) 2020<br>( ) 2021<br>( ) 2022
Language	( ) English<br>( ) French<br>( ) Germain<br>( ) Portuguese<br>( ) Spanish<br>( ) Other
Journal	
Aims of study	
Setting	
Study design	
Follow up of study duration	
Population / participants	
Intervention	
Outcomes and outcomes measurements	
Method of data analysis	
Form completed	( ) Yes<br>( ) No

Annex S4 – Critical Appraisal Tool - GRADE CERQual

Record ID	
Methodological limitation – study design or conducted	( ) No problems<br>( ) Partial problems<br>( ) Many problems<br>( ) Unclear / not applicable (problems in design or conduct of the primary studies supporting a review finding)
Methodological limitation - participants and setting selection	( ) No problems<br>( ) Partial problems<br>( ) Many problems<br>( ) Unclear / not applicable (how and if the selection is described)
Methodological limitation - researcher flexibility	( ) No problems<br>( ) Partial problems<br>( ) Many problems<br>( ) Unclear / not applicable (if the author commented the findings method)
Methodological limitation - data	( ) No problems<br>( ) Partial problems<br>( ) Many problems<br>( ) Unclear / not applicable (how data was collected and analyzed)
Methodological limitation - adequacy of the tool used in the search	( ) No problems<br>( ) Partial problems<br>( ) Many problems<br>( ) Unclear / not applicable (agreement of the best tool)
Methodological limitation - impact	( ) No problems<br>( ) Partial problems<br>( ) Many problems<br>( ) Unclear / not applicable (method impact on the review findings)
Methodological limitation - contribution	( ) No problems<br>( ) Partial problems<br>( ) Many problems<br>( ) Unclear / not applicable (relative contribution of limited studies)
Methodological limitation - classification	( ) No or very minor concerns<br>( ) Minor concerns<br>( ) Moderate concerns<br>( ) Serious concerns
Method assessment	
Coherence – phenomenon of interest	( ) High coherence<br>( ) Partial coherence<br>( ) Low coherence<br>( ) No coherence
Coherence – findings	( ) High coherence<br>( ) Partial coherence<br>( ) Low coherence<br>( ) No coherence
Coherence – data	( ) High coherence<br>( ) Partial coherence<br>( ) Low coherence<br>( ) No coherence
Coherence – classification	( ) No or very minor concerns<br>( ) Minor concerns<br>( ) Moderate concerns<br>( ) Serious concerns

Coherence – assessment	
Adequacy – qualitative data	( ) High adequacy<br>( ) Partial adequacy<br>( ) Low adequacy<br>( ) No adequacy
Adequacy – quantitative data	( ) High adequacy<br>( ) Partial adequacy<br>( ) Low adequacy<br>( ) No adequacy
Adequacy – classification	( ) No or very minor concerns<br>( ) Minor concerns<br>( ) Moderate concerns<br>( ) Serious concerns
Adequacy – assessment	
Relevance – time	( ) High relevance<br>( ) Partial relevance<br>( ) Indirect relevance<br>( ) Low relevance<br>( ) No relevance<br>( ) Unclear / not applicable
Relevance – setting and place	( ) High relevance<br>( ) Partial relevance<br>( ) Indirect relevance<br>( ) Low relevance<br>( ) No relevance<br>( ) Unclear / not applicable
Relevance –topic of interest / intervention	( ) High relevance<br>( ) Partial relevance<br>( ) Indirect relevance<br>( ) Low relevance<br>( ) No relevance<br>( ) Unclear / not applicable
Relevance – population	( ) High relevance<br>( ) Partial relevance<br>( ) Indirect relevance<br>( ) Low relevance<br>( ) No relevance<br>( ) Unclear / not applicable
Relevance – classification	( ) No or very minor concerns<br>( ) Minor concerns<br>( ) Moderate concerns<br>( ) Serious concerns
Relevance – assessment	
OVERAL ASSESSMENT	( ) High confidence<br>( ) Moderate confidence<br>( ) Low confidence<br>( ) Very low confidence

Annex S5 – Quantitative data extraction

	Question 1	Question 2	Question 3	Question 4
Total	1,644	1,063	1,090	465
Disagreement	116	77	53	28
Included	26	37	14	85
Excluded	1,638	1,026	282	380
Reasons for exclusion				
wrong outcome	779	231	171	101
wrong population	431	782	126	216
wrong context	138	115	50	65
wrong study design	68	125	17	21
wrong intervention	213	0	6	0
wrong background	0	568	0	0

Annex S6 – Excluded articles and reasons for exclusion.

Author	Title	Reason for exclusion
Abdel-Qader, D. H.; Hayajneh, W.; Albassam, A.; Obeidat, N. M.; Belbeisi, A. M.; Al Mazrouei, N.; El-Shara, A.A.; El Sharu, H.; Mohammed Ebaed, S.B.; Mohamed Ibrahim, O. [38]	Pharmacists-physicians collaborative intervention to reduce vaccine hesitancy and resistance: A randomized controlled trial	The article is unclear. The results on prevalence are mixed with results of the intervention.
Salmon, D.; Opel, D.J.; Dudley, M.Z.; Brewer, J.; Breiman, R. [18]	Reflections On Governance, Communication, And Equity: Challenges and Opportunities In COVID-19 Vaccination	Study design
Berry, S.D.; Goldfeld, K.S.; McConeghy, K.; Gifford, D.; Davidson, H.E.; Han, L.; Syme, M.; Gandhi, A.; Mitchell, S.L.; Harrison, J.; et al. [39]	Evaluating the Findings of the IMPACT-C Randomized Clinical Trial to Improve COVID-19 Vaccine Coverage in Skilled Nursing Facilities	The comparison is between similar groups, but the study´s development is not clear. The findings are not consistent with the objectives.
Frati, P.; La Russa, R.; Di Fazio, N.; Del Fante, Z.; Delogu, G.; Fineschi, V. [40]	Compulsory Vaccination for Health care Workers in Italy for the Prevention of SARS-CoV-2 Infection	Opinion report
Hughes, K.; Gogineni, V.; Lewis, C.; Deshpande, A. [41]	Considerations for fair prioritization of COVID-19 vaccine and its mandate among Health care personnel	Opinion report
Beekmann, S.E.; Babcock, H.M.; Rasnake, M.S.; Talbot, T.R.; Polgreen, P.M. [42]	Coronavirus disease 2019 (COVID-19) vaccination preparedness policies in US hospitals	The article is original, but the method does not provide details on the research steps.
Cukier, A. [43]	COVID-19 Update: Saskatchewan intensive care crisis, new vaccine safety data, and pushback on health worker mandates	News article
Gur-Arie, R.; Berger, Z.; Rubinstein, R.D. [44]	COVID-19 Vaccine Uptake Through the Lived Experiences of Health Care Personnel: Policy and Legal Considerations	Literature review without a defined methodology
de Almeida, B.G.; Massa, C.O.B.; de Souza, J.P.; de Paula Moura, L.; Pinto, P.M.R.V.; Broucke, V.R.B.V. [45]	Uma dose de esperança: o processo de vacinação dos trabalhadores da saúde	Qualitative study without an appropriate methodology
Royal Australian College of General Practitioners [46]	Mandatory COVID-19 vaccination for Health care workers including GPs	Opinion article

Annex S7 – General/target population - data extraction from included studies.

Author, Year	Country	Title	Language	Journal	Study Design	Sample (population or studies)
**Study that addressed population intention for vaccination and refusal cause**						
Author, Year	Country	Title	Language	Journal	Study Design	Sample (population or studies)
Zheng, H.; Jiang, S.; Wu, Q., 2022 [19]	USA	Factors influencing COVID-19 vaccination intention: The roles of vaccine knowledge, vaccine risk perception, and doctor-patient communication	English	Patient Education and Counseling	Cross-sectional	800
**Studies that addressed population acceptance, barriers, and access to vaccines**						
Author, Year	Country	Title	Language	Journal	Study Design	Sample (population or studies)
Abba-Aji, M.; Stuckler, D.; Galea, S.; McKee, M., 2022 [29]	Turkey	Ethnic/racial minorities' and migrants' access to COVID-19 vaccines: A systematic review of barriers and facilitators	English	Journal of Migration and Health	Systematic review	33 studies
Al-Metwali, B.; Al-Jumaili, A.Z.; Al-Alag, Z.S.B., 2022 [10]	Israel	Exploring the acceptance of the COVID-19 vaccine among health care workers and the general population using the health belief model	English	J Evaluation in Clinical Practice	Cross-sectional	1680
**Studies that addressed strategies for covering the population**						
Author, Year	Country	Title	Language	Journal	Study Design	Sample (population or studies)
Leila, R.A.; Salamah, M.; El-Nigoumi, S., 2021 [28]	Bahrain	Reducing COVID-19 Vaccine Hesitancy by Implementing Organizational Intervention in a Primary Care Setting in Bahrain	English	Curreus	Interviews	193

Study that addressed population intention for vaccination and refusal cause						
Author, Year	Country	Title	Language	Journal	Study Design	Sample (population or studies)
Zheng, H.; Jiang, S.; Wu, Q., 2022 [19]	USA	Factors influencing COVID-19 vaccination intention: The roles of vaccine knowledge, vaccine risk perception, and doctor-patient communication	English	Patient Education and Counseling	Cross-sectional	-
**Studies that addressed population acceptance, barriers, and access to vaccines**						
Author, Year	Country	Title	Language	Journal	Study Design	Sample (population or studies)
Abba-Aji, M.; Stuckler, D.; Galea, S.; McKee, M., 2022 [29]	Turkey	Ethnic/racial minorities' and migrants' access to COVID-19 vaccines: A systematic review of barriers and facilitators	English	Journal of Migration and Health	Systematic review	-
Al-Metwali, B.; Al-Jumaili, A.Z.; Al-Alag, Z.S.B.2022 [10]	Israel	Exploring the acceptance of the COVID-19 vaccine among health care workers and the general population using the health belief model	English	J Evaluation in Clinical Practice	Cross-sectional	-
**Studies that addressed strategies for covering the population**						
Leila, R.A.; Salamah, M.; El-Nigoumi, S., 2021 [28]	Bahrain	Reducing COVID-19 Vaccine Hesitancy by Implementing Organizational Intervention in a Primary Care Setting in Bahrain	English	Curreus	Interviews	

*Participants are both population and HCW

Annex S8 –Health Care workers - data extraction from included studies.

Author, Year	Country	Title	Language	Journal	Study Design	Sample (population or studies)
Studies that address HCW intention for vaccination, acceptance and refusal motivation						
Zheng, H.; Jiang, S.; Wu, Q., 2022 [19]	USA	Factors influencing COVID-19 vaccination intention: The roles of vaccine knowledge, vaccine risk perception, and doctor-patient communication	English	Patient Education and Counseling	Cross-sectional	800
Al-Metwali, B.; Al-Jumaili, A.Z.; Al-Alag, Z.S.B., 2022 [10]	Israel	Exploring the acceptance of the COVID-19 vaccine among healthcare workers and the general population using the health belief model	English	J Evaluation in Clinical Practice	Cross-sectional	1680
Studies that address HCW Attitudes towards vaccination						
Manby, L.; Dowrick, A.; Karia, A.; Maio, L.; Buck, C.; Singleton, G.; Lewis-Jackson, S.; Uddin, I.; Vanderslott, S.; Martin, S.; et al., 2022 [34]	UK	Healthcare workers' perceptions and attitudes towards the UK's COVID-19 vaccination program: a rapid qualitative appraisal	English	BMJ Open	Interviews	48
Aci, O.S.; Kackin, O.; Karaaslan, S.; Ciydem, E., 2022 [20]	Turkey	Qualitative examination of the attitudes of healthcare workers in Turkey regarding COVID-19 vaccines	English	International Journal of Nursing Knowledge	Interviews	36
Poon, P.K.M.; Zhou, W.; Chan, D.C.C.; Kwok, K.O.; Wong, S.Y.S., 2021 [35]	China (Hong Kong)	Recommending COVID-19 Vaccines to Patients: Practice and Concerns of Frontline Family Doctors	English	Vaccines	Cross-sectional	312
Li, M.; Luo, Y.; Watson, R.; Zheng, Y.; Ren, J.; Tang, J.; Chen, Y., 2021 [25]	USA	Healthcare workers' (HCWs) attitudes and related factors towards COVID-19 vaccination: A rapid systematic review	English	Post Grade Med J	Systematic review	13 studies

Carpenter, D.M.; Hastings, T.; Westrick, S.; Mashburn, P.; Rosenthal, M.; Smith, M.; Kiser, S.; Gamble, A.; Brewer, N.T.; Curran, G., 2022 [23]	USA	Rural community pharmacists' ability and interest in administering COVID-19 vaccines in the Southern United States	English	Journal of the American Pharmacists Association	Cross-sectional	69
Studies that addressed HCW vaccination hesitancy reasons						
Toth-Manikowski, S.M.; Swirsky, E.S.; Gandhi, R.; Piscitello, G., 2022 [27]	USA	COVID-19 vaccination hesitancy among health care workers, communication, and policy-making	English	American Journal of Infection Control	Cross-sectional	1974
Qunaibi, E.; Basheti, I.; Soudy, M.; Sultan, I., 2021 [7]	Multinational	Hesitancy of Arab Healthcare Workers towards COVID-19 Vaccination: A Large-Scale Multinational Study	English	Vaccines	Cross-sectional	5708
Ashok, N.; Krishnamurthy, K.; Singh, K.; Rahman, S.; Majumder, M.A.A., 2021 [30]	India	High COVID-19 Vaccine Hesitancy Among Health care Workers: Should Such a Trend Require Closer Attention by Policymakers?	English	Curreus	Cross-sectional	264
Harrison, J.; Berry, S.; Mor, V.; Gifford, D., 2021 [26]	USA	"Somebody Like Me": Understanding COVID-19 Vaccine Hesitancy among Staff in Skilled Nursing Facilities	English	Journal of the American Medical Directors Association	Focus Group	58
Studies that addressed strategies to support HCW vaccination						
Giannitrapani, K.F.; Brown-Johnson, C.; Connell, N.B.; Yano, E.M.; Singer, S.J.; Giannitrapani, S.N.; Thanassi, W.; Lorenz, K.A., 2022 [36]	USA	Promising Strategies to Support COVID-19 Vaccination of Healthcare Personnel: Qualitative Insights from the VHA National Implementation	English	Journal of General Internal Medicine	Interviews	43
Kim, M.H.; Son, N.H.; Park, Y.S.; Lee, J.H.; Kim, D.A.; Kim, Y.C., 2021 [24]	Korea	Effect of a hospital-wide campaign on COVID-19 vaccination uptake among healthcare workers in the context of raised concerns for life-threatening side effects	English	Plos One	Cross-sectional	837
Studies that addressed policies for covering HCW vaccination						

Pitini, E.; Baccolini, V.; Rosso, A.; Massimi, A.; De Vito, C.; Marzuillo, C.; Villari, P., 2021 [21]	Italy	How Public Health Professionals View Mandatory Vaccination in Italy—A Cross-Sectional Survey	English	Vaccines	Cross-sectional	1350
Riccò, M.; Ferraro, P.; Peruzzi, S.; Balzarini, F.; Ranzieri, S., 2021 [9]	Italy	Mandate or Not Mandate: Knowledge, Attitudes, and Practices of Italian Occupational Physicians towards SARS-CoV-2 Immunization at the Beginning of Vaccination Campaign	English	Vaccines	Cross-sectional	166
Baumer-Mouradian, S.H.; Collins, S.; Lausten, T.; Pohl, C.; Sisney, M.; Khare, S.; Ose, M.; Roe, J.; Reilly, C.; Gutzeit, M., 2021 [37]	USA	Urgent COVID-19 Vaccination of Healthcare Workers via a Quality Improvement Initiative	English	Pediatric Quality & Safety	Cross-sectional	3921
Turbat, B.; Sharavyn, B.; Tsai, F.-J., 2022 [22]	Mongolia	Attitudes towards Mandatory Occupational Vaccination and Intention to Get COVID-19 Vaccine during the First Pandemic Wave among Mongolian Healthcare Workers: A Cross-Sectional Survey	English	International Journal of Environmental Research and Public Health	Cross-sectional	238

*Participants are both population and HCW

Annex S9 - List of the general or target population studies´ findings and details of the GRADE CERQual assessment.

Author/Year	Title	Study design	Findings	Evidence	Summary assessment
Zheng, H.; Jiang, S.; Wu, Q., 2022 [19]	Factors influencing COVID-19 vaccination intention: The roles of vaccine knowledge, vaccine risk perception, and doctor-patient communication	Cross sectiona l	First, susceptibility to COVID-19 vaccine side effects was negatively associated with vaccination intention, whereas perceived severity did not show any significant impact. Second, vaccine-related knowledge was not directly related to vaccination intention, but it had an indirect and positive effect on vaccination intention via decreasing perceived susceptibility. Third, doctor-patient communication strengthened the negative effect of vaccine knowledge on perceived susceptibility and severity. Practical implications: Government agencies should actively emphasize the effectiveness and importance of vaccination.	Moderate Confidenc e	In this cross-sectional study, the criteria for inclusion in the sample were clear. Participants were recruited from an online panel managed by the survey company. The sample was small, and quota sampling was used to ensure that the distribution reflected the national profile. Only people with internet access were able to participate in the study. Using an online tool, the study was able to include more participants. The findings, data, and phenomena were coherent. The study was developed at a relevant time, in the early stage of vaccination programs in the United States. The study proposed a nationwide vision but presents a limitation in the representativeness of the participants. The strategy can be used by other researchers, and the results support vaccination uptake. The main findings show evidence regarding the impact of perceived susceptibility to COVID-19 vaccine side effects on vaccination intention in a sample of American adults. The findings also suggest that health professionals could help reduce the perceived risk associated with vaccine´s side effects in the public at large. Another important finding is doctor-patient communication, which plays a crucial role in facilitating vaccination intention.
Abba-Aji, M.; Stuckler, D.; Galea, S.; McKee, M., 2022 [29]	Ethnic/racial minorities' and migrants' access to COVID-19 vaccines: A systematic review of barriers and facilitators	Systema tic review	Of a total of 248 studies screened, 33 met the criteria and were included in the final sample. 31 of the included studies were conducted in high-income countries, including USA (n = 17 studies), UK (n = 10), Qatar (n = 2), Israel (n = 1), and France (n = 1). One study was in an upper middle-income country, China (n = 1), and another covered multiple country (n = 1). 26 studies reported outcomes for ethnic minorities, while 9 studies reported on migrants. Most of the studies were quantitative cross-sectional (n = 24) and ecological studies (n = 4). The remaining were	High confidence	The study is about migrants' access to vaccination in many countries. It consists of a systematic review with a clear methodology and coherence, adequacy, and relevance in all points of the assessment. The study is clear, rich in details, and useful for government politics.

			qualitative (n = 4) and mixed methods (n = 1). There was consistent evidence of high levels of COVID-19 vaccine hesitancy among Black/Afro-Caribbean groups in the USA and UK, while studies of Hispanic/Latino populations in the USA and Asian populations in the UK provided mixed evidence, with levels that were higher, lower, or the same as their White counterparts. Asians in the USA had the highest COVID-19 vaccine acceptance compared to other ethnic groups. There was higher vaccine acceptance among migrant groups in Qatar and China than in the general population. However, migrants to the UK experienced barriers to vaccine access, mainly attributed to language and communication issues. Lack of confidence, mainly due to mistrust of government and health systems coupled with poor communication were the main barriers to vaccine uptake among Black ethnic minorities and migrants.		
Al-Metwali, B.; Al-Jumaili, A.Z.; Al-Alag, Z.S.B., 2021 [10]	Exploring the acceptance of the COVID-19 vaccine among healthcare workers and the general population using the health belief model	Cross sectional	A total of 1,680 completed surveys were received. Mean age was 31.2 ± 9.9 years, with 53.0% females and 47.0% males. The largest group was HCWs (45.7%), followed by the general population (37.5%) and university students in health fields (16.8%). The findings expressed some hesitancy to COVID-19 vaccination, with an acceptance rate of 61.7%. HCWs perceived significantly higher susceptibility and severity of COVID-19 infection compared to the general population. HCWs were significantly more likely than the general population to receive the COVID-19 vaccine. Concerns with proper storage were the main barrier to receiving the vaccine. Regression analysis indicated eight factors significantly associated with willingness to receive the COVID-19 vaccine: Preventive measures, perceived benefit, perceived barriers, cue to action, subjective norm, support for vaccination in general, and having received a flu vaccine previously.	High confidence	The method is clear, and the researchers used an appropriate tool. The HBM successfully predicted the factors influencing people's acceptance of vaccination against COVID-19. Coherence between the target phenomenon, data, and findings. Data are consistent with the interest described in the findings. The sample size is small, and most of the respondents were from Baghdad, not representing the entire population of Iraq.
Leila, R.A.; Salamah, M.; El-Nigoumi, S., 2021 [28]	Reducing COVID-19 Vaccine Hesitancy by Implementing Organizational Intervention in a Primary Care Setting in Bahrain	Interviews	There were 665 hesitant patients before the intervention. However, after the intervention, the number decreased to 193 patients, and the control chart revealed a reliable process. The percentage of recommendations by physicians increased from 1% to 51% after 19 weeks of implementation and with a controlled process. Rectifying process barriers and upgrading physicians' skills would improve the COVID-19 vaccine counseling rate, and tailored communication would reduce the hesitancy rate. Nevertheless, the study was constrained by lack of information on the impact of social media and national measures on patients' decisions.	High confidence	The method's development is clear and well done. The study does not present methodological limitations. It is well designed, the participants' selection is clear, and data collection and analysis follow best practices. The study has no ambiguity in the data, and the findings are clear. Both qualitative and quantitative data are clear and detailed. The theme is relevant and developed coherently.

*Participants are both population and HCW

Annex S10 - List of the HCW studies´ findings and details of the GRADE CERQual assessment.

Author/Year	Title	Study design	Findings	Evidence	Summary assessment
Zheng, H.; Jiang, S.; Wu, Q., 2022 [19]	Factors influencing COVID-19 vaccination intention: The roles of vaccine knowledge, vaccine risk perception, and doctor-patient communication	Cross-sectional	First, susceptibility to COVID-19 vaccine side effects was negatively associated with vaccination intention, whereas perceived severity did not show any significant impact. Second, vaccine-related knowledge was not directly related to vaccination intention, but it had an indirect and positive effect on vaccination intention via decreasing perceived susceptibility. Third, doctor-patient communication strengthened the negative effect of vaccine knowledge on perceived susceptibility and severity. Practical implications: Government agencies should actively emphasize the effectiveness and importance of vaccination.	Moderate Confidence	In this cross-sectional study, the criteria for inclusion in the sample were clear. Participants were recruited from an online panel managed by the survey company. The sample was small, and quota sampling was used to ensure that the distribution reflected the national profile. Only people with internet access were able to participate in the study. Using an online tool, the study was able to include more participants. The findings, data, and phenomena were coherent. The study was developed at a relevant time, in the early stage of vaccination programs in the United States. The study proposed a nationwide vision but presents a limitation in the representativeness of the participants. The strategy can be used by other researchers, and the results support vaccination uptake. The main findings show evidence regarding the impact of perceived susceptibility to COVID-19 vaccine side effects on vaccination intention in a sample of American adults. The findings also suggest that health professionals could help reduce the perceived risk associated with vaccine´s side effects in the public at large. Another important finding is doctor-patient communication, which plays a crucial role in facilitating vaccination intention.

Abba-Aji, M.; Stuckler, D.; Galea, S.; McKee, M., 2022 [29]	Ethnic/racial minorities' and migrants' access to COVID-19 vaccines: A systematic review of barriers and facilitators	Systematic review	Of a total of 248 studies screened, 33 met the criteria and were included in the final sample. 31 of the included studies were conducted in high-income countries, including USA (n = 17 studies), UK (n = 10), Qatar (n = 2), Israel (n = 1), and France (n = 1). One study was in an upper middle-income country, China (n = 1), and another covered multiple country (n = 1). 26 studies reported outcomes for ethnic minorities, while 9 studies reported on migrants. Most of the studies were quantitative cross-sectional (n = 24) and ecological studies (n = 4). The remaining were qualitative (n = 4) and mixed methods (n = 1). There was consistent evidence of high levels of COVID-19 vaccine hesitancy among Black/Afro-Caribbean groups in the USA and UK, while studies of Hispanic/Latino populations in the USA and Asian populations in the UK provided mixed evidence, with levels that were higher, lower, or the same as their White counterparts. Asians in the USA had the highest COVID-19 vaccine acceptance compared to other ethnic groups. There was higher vaccine acceptance among migrant groups in Qatar and China than in the general population. However, migrants to the UK experienced barriers to vaccine access, mainly attributed to language and communication issues. Lack of confidence, mainly due to mistrust of government and health systems coupled with poor communication were the main barriers to vaccine uptake among Black ethnic minorities and migrants.	High confidence	The study is about migrants' access to vaccination in many countries. It consists of a systematic review with a clear methodology and coherence, adequacy, and relevance in all points of the assessment. The study is clear, rich in details, and useful for government politics.
Al-Metwali, B.; Al-Jumaili, A.Z.; Al-Alag, Z.S.B., 2021 [10]	Exploring the acceptance of the COVID-19 vaccine among healthcare workers and the general population using the health belief model	Cross-sectional	A total of 1,680 completed surveys were received. Mean age was 31.2 ± 9.9 years, with 53.0% females and 47.0% males. The largest group was HCWs (45.7%), followed by the general population (37.5%) and university students in health fields (16.8%). The findings expressed some hesitancy to COVID-19 vaccination, with an acceptance rate of 61.7%. HCWs perceived significantly higher susceptibility and severity of COVID-19 infection compared to the general population. HCWs were significantly more likely than the general population to receive the COVID-19 vaccine. Concerns with proper storage were the main barrier to receiving the vaccine. Regression analysis indicated eight factors significantly associated with willingness to receive the COVID-19 vaccine: Preventive measures, perceived benefit, perceived barriers, cue to action, subjective norm, support for vaccination in general, and having received a flu vaccine previously.	High confidence	The method is clear, and the researchers used an appropriate tool. The HBM successfully predicted the factors influencing people's acceptance of vaccination against COVID-19. Coherence between the target phenomenon, data, and findings. Data are consistent with the interest described in the findings. The sample size is small, and most of the respondents were from Baghdad, not representing the entire population of Iraq.
Manby, L.; Dowrick, A.; Karia, A.; Maio, L.; Buck, C.; Singleton, G.;	Healthcare workers' perceptions and attitudes towards the UK's COVID-19 vaccination programme: a rapid qualitative appraisal	Interviews	Uncertainty about the long-term safety of vaccines and efficacy against mutant strains made it difficult for HCWs to balance the benefits against the risks of vaccination. HCWs felt that government decisions on vaccine rollout had not been supported by evidence-based science, and this impacted their level of trust and confidence in the program. The online spread of misinformation also impacted	Moderate Confidence	The aim was to provide a nationwide study. The sample is small and does not represent the entire country. Few junior-level and Black, Asian, and minority ethnic (BAME) HCWs were interviewed. The sample and setting are not consistent with the objective. The study is highly coherent, and the

Lewis-Jackson, S.; Uddin, I.; Vanderslott, S.; Martin, S.; et al., 2022 [34]			HCWs' attitudes towards vaccination, particularly among junior-level and black, Asian, and minority ethnic (BAME) HCWs. Most HCWs felt encouraged to promote vaccination for their patients, and the majority said they would advocate vaccination or engage in conversations about vaccination with others when relevant.		adequacy is suitable. HCWs are the focus of the study. However, the nationwide scope was not represented, with only 2 Health care facilities.
Aci, O.S.; Kackin, O.; Karaaslan, S.; Ciydem, E., 2022 [20]	A qualitative examination of the attitudes of Health care workers in Turkey regarding COVID-19 vaccines	Interviews	Most healthcare workers participating in the study were male and married, with an average age of 34 ± 19 years. In addition, 52.8% participants worked in a state hospital, the duration of their working lives was 11.31 ± 7.95 years, and the duration of their providing care to patients diagnosed with COVID-19 was 4.25 ± 5.73 months. Turkish healthcare workers´ attitudes toward the COVID-19 vaccine were divided into three themes: "influencing factors," "priority group", and "trust." Vaccine hesitancy can be addressed by careful attention to the application of vaccination programs, correct and effective use of social media, transparent, and precise management of political processes, and the provision of evidence-based information about the vaccines	High confidence	The study is methodologically complete, with an in-depth approach and relevant theme. The study does not present methodological limitations. The design is adequate, participants' selection is clear, and data collection and analysis follow best practices. The study tool is also suitable for the propose. The method directly impacts the findings. The study is coherent since the findings reflect the COVID-19 vaccination phenomenon, with no ambiguity in the data. The study is rich in detail and the sample is suitable. The study sample is nationwide. Although the search lasted 13 days, the findings were well conducted.
Poon, P.K.M.; Zhou, W.; Chan, D.C.C.; Kwok, K.O.; Wong, S.Y.S., 2021 [35]	Recommending COVID-19 Vaccines to Patients: Practice and Concerns of Frontline Family Doctors	Cross-sectional	A total of 312 family doctors responded (17.6% response rate). The proportion of doctors who had received COVID-19 vaccines was 90.1%. The proportion of doctors who would recommend vaccination of all patients without contraindications was 64.4%. The proportion of doctors who would proactively discuss COVID-19 vaccines with patients was 52.9%. Multivariate logistic regression showed that doctors' COVID-19 vaccination status was the strongest predictor of family doctors making a recommendation to patients. Longer duration of medical practice, willingness to initiate the relevant discussion with patients, and less worry about vaccine side effects in patients with chronic illnesses were the other factors associated with making a COVID-19 vaccination recommendation. Family doctors should be encouraged to get vaccinated themselves and initiate discussions about COVID-19 vaccines with patients. Vaccine safety data on patients with chronic illnesses, training, and guidelines for junior doctors may facilitate COVID-19 vaccination recommendations by family doctors.	High confidence	The study approaches the value of family physicians' recommendations and vaccination results in the target population. The design is a cross-sectional online anonymous survey. The study was developed in the second year of the COVID-19 pandemic. Participants were members and fellows of the Hong Kong College of Family Physicians, the sole governing body for the professional training of family medicine specialists in HK. The setting and family physicians are relevant in the second pandemic year for reaching the target population. The tools were suitable for the study design. Data are deeply explained. The findings are clear and coherent with the target phenomenon, as are the outcome data.
Turbat, B.; Sharavyn, B.; Tsai, F.-J., 2022 [22]	Attitudes towards Mandatory Occupational Vaccination and Intention to Get COVID-19 Vaccine during the First Pandemic Wave among Mongolian Healthcare Workers: A Cross-Sectional Survey	Cross-sectional	While only 39.9% of HCWs were aware of recommended occupational vaccinations, they strongly agreed with mandatory occupational vaccination for HCWs (93.7%). The agreement rate was significantly higher than their attitude toward general vaccination (93.7% vs. 77.8%). HCW's willingness to get the COVID-19 vaccine was high (67.2%). HCWs aged 26-35 years who worked in tertiary hospitals were less willing to get the COVID-19 vaccine (50%). Participants with lower confidence in the efficacy of the COVID-19	Moderate confidence	The design was a cross-sectional study based on an online survey with a convenience sample. A total of 1,576 HCWs viewed the questionnaire, but only 238 responded, showing a low response rate. The study design is a self-reported survey. The survey only approaches WHO and Ministry of Health recommendations on occupational vaccines for measles, polio, rubella, pertussis, influenza, BCG,

			vaccine and less positive attitudes toward general vaccination were less likely to get the COVID-19 vaccine. Agreement to mandatory occupational vaccination was higher in Mongolia than in other countries. Intention to get the COVID-19 vaccine was high and associated with confidence in the vaccine´s effectiveness.		hepatitis B, varicella, diphtheria, meningococcal meningitis, tuberculosis, and tetanus. There was no question on mandatory COVID-19vaccination. The findings on intent to be vaccinated were consistent with the data and COVID- 19 phenomenon. The data are sufficiently detailed for assessment purposes. It is not clear whether the sample represents the country's entire reality. A subset is available as a population.
Toth-Manikowski, S.M.; Swirsky, E.S.; Gandhi, R.; Piscitello, G., 2022 [27]	COVID-19 vaccination hesitancy among health care workers, communication, and policy-making	Cross-sectional	In 1,974 completed responses, 85% of HCWs received or anticipated receiving COVID-19 vaccination. Multivariate logistic regression found HCWs were less likely to receive COVID-19 vaccination if they were Black, Republican, or allergic to any vaccine component and more likely to receive it if they believed people close to them thought it was important for them to receive the vaccine. A sizable number remained vaccine-hesitant 1 year into the COVID-19 pandemic. As HCWs are positively influenced by colleagues who believe in COVID-19 vaccination, the development of improved communication across HCW departments and roles may improve vaccination rates.	High<br><br>Confidence	The study asks HCWs about being vaccinated and mandatory vaccination. There are recommendations to improve HCW vaccination and professional skills. The COVID-19 Vaccine Attitude Scale was adapted from a questionnaire previously developed to measure behavioral determinants of vaccine uptake among healthcare workers during the H1N1 influenza pandemic. The predictive variables used in the multivariate regression analysis are complete and consistent with the findings. The qualitative and quantitative data are detailed and appropriate for the method. The study is relevant, coherent, and adequate, and has no methodological limitations.
Giannitrapani, K.F.; Brown-Johnson, C.; Connell, N.B.; Yano, E.M.; Singer, S.J.; Giannitrapani, S.N.; Thanassi, W.; Lorenz, K.A., 2022 [36]	Promising Strategies to Support COVID-19 Vaccination of Healthcare Personnel: Qualitative Insights from the VHA National Implementation	Interviews	Participants were 22 physicians, 17 nurse practitioners and physician assistants, and 4 registered nurses, from 29 of VHA medical centers throughout the USA. Specifically, the themes included the following: (1) use interdisciplinary task forces to leverage diverse skillsets for vaccine implementation; (2) create detailed processes, addressing time trade-offs for personnel involved in vaccine clinics, designating process/authority to shift personnel where needed, and proactively involving leaders to support resource allocation/alignment; (3) expect and accommodate vaccine buy-in occurring over time: prepare for some HCP's slow buy-in, align buy-in facilitation with identities and motivation, and encourage word-of-mouth and hyper-local testimonials; (4) overcome misinformation with trustworthy communication (5) use existing and newly developed communication channels to foster shared learning across teams and sites.	High confidence	The method is explained in clear detail. There are no limitations to the methodology. The data are consistent with the phenomenon and the findings. The issue, participants, and findings are relevant and representative. The study is useful for policymakers to improve the health system's response through COVID-19 vaccination.
Carpenter, D.M.; Hastings, T.; Westrick, S.; Mashburn, P.; Rosenthal, M.; Smith, M.; Kiser, S.; Gamble, A.;	Rural community pharmacists' ability and interest in administering COVID-19 vaccines in the Southern United States	Cross-sectional	69 of 106 pharmacists completed the survey (response rate = 65%). Approximately half of the pharmacists were ready (52%) or actively taking steps (39%) to provide COVID-19 vaccines in the next 6 months. Pharmacies had a median of 2 staff members who were authorized to administer COVID-19 vaccines. Almost half (46%) estimated they could administer more than 30 doses of vaccine per day. Most pharmacies could store vaccines at standard refrigeration (90%) and freezing (83%) levels needed for thawed and premixed vaccines, respectively. Most pharmacists planned to access COVID-19	High Confidence	The method is explained in clear detail. Participants´ responses may have been biased by the $25 stipend. The authors discuss the inclusion of pharmacists in vaccination campaigns. The data are coherent, and the findings reflect the target phenomenon. The data are consistent with the phenomenon and findings. The issue, participants, and findings are relevant and representative.

Brewer, N.T.; Curran, G., 2022 [23]			vaccines through an agreement with a state or local public health entity (48%) or by ordering through group purchasing organizations (46%). Only 23% of pharmacists had received any vaccine training, and only 48% were highly motivated to receive the vaccine themselves.		
Harrison, J.; Berry, S.; Mor, V.; Gifford, D., 2021 [26]	"Somebody Like Me": Understanding COVID-19 Vaccine Hesitancy among Staff in Skilled Nursing Facilities	Focus group	The findings indicated that some Skill Nursing Facilities' staff were hesitant to receive the COVID-19 vaccine. Reasons for this hesitancy included beliefs that the vaccine was developed too quickly and without sufficient testing; personal fears about preexisting medical conditions, and more general distrust of the government. Nursing' staff indicated that seeing people like themselves receive the vaccination was more important than seeing public figures. The study cited vaccination effort as a social enterprise and the need to develop long-term care provider-academic-community partnerships in response to COVID-19 and expectation of future pandemics.	Low confidence	Focus group data were analyzed using a phenomenological approach featuring open-ended questions to understand staff experiences with COVID-19 testing and vaccination. The sample size (n =58) is small relative to the 4.5 million healthcare workers in the USA. The authors reflect superficially on the findings. The analysis is not clear or adequately discussed. The method is not able to be replicated to strengthen the study´s impact. Some findings are useful for organizing media campaign policies. The discussion is lost in the demographic analysis, with racial issues revealing insufficient selection of participants. Staff preference to see local community members and people like themselves be vaccinated to improve their confidence is superficial in a discussion about vaccine acceptance during a pandemic. The interview asked about participants' acceptance in three aspects: vaccine availability, reasons for refusing the vaccine, and a remote possibility of a changed scenario. Quantitative and qualitative data are not adequate. The setting was consistent with the objectives. The results are superficially described and fail to explain the context.
Kim, M.H.; Son, N.H.; Park, Y.S.; Lee, J.H.; Kim, D.A.; Kim, Y.C., 2021 [24]	Effect of a hospital-wide campaign on COVID-19 vaccination uptake among healthcare workers in the context of raised concerns for life-threatening side effects	Cross-sectional	A tailored intervention strategy based on a survey can improve COVID-19 vaccination uptake. Of 1,171 HCWs who had received the first dose of the vaccine, 71.5% completed the online survey, of whom 3.7% refused to take the second dose and 22.3% showed hesitancy. Hesitancy to receive a second dose was significantly associated with age under 30 years and was less common among those who trusted the effectiveness and safety of the vaccine. Among HCWs who received the first dose, 96.2% completed vaccination with the second dose. Of those who answered the questionnaire and were asked about the timing of their decision to receive the second dose, 57.1% reported that they were motivated by the hospital-wide campaign.	High Confidence	The method is explained in clear detail. The survey was applied in two phases, addressing the acceptance of both the first and second doses. The measures applied are described well, with in-depth discussion of the approaches. There are no limitations to the methodology. The study is coherent, and the data are consistent with the phenomenon and findings. The qualitative and quantitative data are adequate. Detailed tables and figures contribute to the explanation. The topic, participants, and findings are relevant and representative. The study is useful for vaccine uptake by the HCW when planning vaccination campaigns.

Qunaibi, E.; Basheti, I.; Soudy, M.; Sultan, I., 2021 [7]	Hesitancy of Arab Healthcare Workers towards COVID-19 Vaccination: A Large-Scale Multinational Study	Cross-sectional	This large-scale multinational post-vaccine-availability study on COVID-19 vaccine hesitancy among HCWs revealed high rates of hesitancy among Arab-speaking HCWs. Unless addressed properly, this hesitancy can impede the efforts for achieving widespread vaccination. The survey recruited 5,708 participants from 21 Arab countries (87.5%) and 54 other countries (12.5%). The analysis showed a significant rate of vaccine hesitancy among HCWs residing in and outside of Arab countries (25.8% and 32.8%, respectively). The highest rates of hesitancy were among participants from Egypt, Morocco, Tunisia, and Algeria. The most widely cited reasons for hesitancy were concerns about side effects, distrust of expedited vaccine production, and healthcare policies. Factors associated with higher hesitancy included age 30-59 years, previous or current suspected or confirmed COVID-19, female gender, not knowing the vaccine type authorized in the respective country, and not regularly receiving the influenza vaccine. Unless addressed properly, this hesitancy can impede efforts for achieving widespread vaccination and collective immunity.	High confidence	A cross-sectional study was conducted through an online survey. There were no methodological limitations. The survey uses an adequate tool. Participants´ selection, settings, and method are described in detail. The analysis is based on all the collected data. The outcomes have an important impact, and the method can be applied in futures studies. The findings are discussed and reflect the target phenomenon. Qualitative and quantitative data are presented in detail to understand the phenomenon described in the findings. The survey was developed in 75 countries with Arab-speaking HCWs, totaling 5,708 respondents. The findings suggest the improvement of communication with HCWs, listening to their concerns and suggestions, with the development of a transparent, evidence-based healthcare policy, and the incorporation of representative healthcare workers in healthcare decision-making.
Ashok, N.; Krishnamurthy, K.; Singh, K.; Rahman, S.; Majumder, M.A.A., 2021 [30]	High COVID-19 Vaccine Hesitancy Among Healthcare Workers: Should Such a Trend Require Closer Attention by Policymakers?	Cross-sectional	The study showed a high rate of vaccine hesitancy (delay /refusal) among HCWs. Among 264 respondents, 40.2% of HCWs would receive the COVID-19 vaccine if available and 32.2% were willing to take the vaccine after observing adverse effects in others. Factors significantly associated with vaccine acceptance were infected members in social networks, COVID-19 knowledge, the safety of vaccines, and not having received a flu vaccine in the previous year. The main reasons for delay/refusal to vaccinate were rapid vaccine development and compromised quality (43.7%) and lack of trusted information regarding COVID-19 (41.3%).	Low confidence	The study is limited to individuals with internet access, which is a major limiation. The sample is also small, with fewer respondents and unequal distribution of resptondents across the country (India). The target phenomenon and findings are relevant, but the data need to be more consistent because the sample is too small. The data are inadequate because the sample includes only physicians, mainly pediatricians. The sample size was too limited to obtain significant predictors of vaccination in multinomial regression. However, the study´s implementation and topic of interest are relevant. The study is intended to be national, but the country´s population appears to be underrepresented. The subset of the population is not representative.
Pitini, E.; Baccolini, V.; Rosso, A.; Massimi, A.; De Vito, C.; Marzuillo, C.; Villari, P. , 2021 [21]	How Public Health Professionals View Mandatory Vaccination in Italy—A Cross-Sectional Survey	Cross-sectional	Among the 1,044 respondents (77% response rate), a large majority favored the Italian mandatory vaccination law (91%) and were against its repeal (74%). According to respondents, maintaining high vaccination coverage without the need for mandatory vaccination would be preferable, and thus efforts to promote vaccine confidence and proactive vaccine uptake are still needed.	Low Confidence	The methodology has some limitations. The impact of the pandemic is more severe than known diseases. So, the comparison is unbalanced, and the contribution is weak for the COVID-19 vaccination mandatory policy. The target phenomenon and findings should be coherent with the COVID-19 context. Qualitative and quantitative data can help understand the main measures for mandatory vaccination for known diseases. The study has no

					relevance for the setting or target topic. It also was conducted according to a law published before the pandemic.
Riccò, M.; Ferraro, P.; Peruzzi, S.; Balzarini, F.; Ranzieri, S., 2021 [9]	Mandate or Not Mandate: Knowledge, Attitudes, and Practices of Italian Occupational Physicians towards SARS-CoV-2 Immunization at the Beginning of Vaccination Campaign	Cross-sectional	Occupational physicians exhibited wide acceptance of SARS-CoV-2/COVID-19 vaccination, and the majority endorsed required vaccination for HCWs, which may help improve vaccination rates in occupational settings. A high perception of SARS-CoV-2 risk was reported in around 80% of participants (79.5% regarding its occurrence, 81.9% regarding its potential severity). Vaccination was endorsed by 90.4% of respondents. Endorsement of required vaccination was reported by 60.2% of respondents.	High confidence	The study is coherent, and the method is clear, complete, and well-designed. Practice by occupational physicians is closely related to the target phenomenon. The authors offer reflections on the relationship between the objectives and findings. Thet study tools were adequate for this type of research. The sample was representative and can contribute to work by other vaccination teams. Regarding adequacy, the issue of mandatory versus nonmandatory vaccination requires further investigation to help health system managers make decisions. The study is relevant because the participants can become critical players in encouraging vaccine acceptance. In addition, although an online tool was used, the authors performed an excellent study with a complete analysis. After the survey, the results showed that occupational physicians showed wide acceptance of COVID-19 vaccination, and the majority endorsed mandatory vaccination for HCWs, which may help improve vaccination rates in occupational settings.
Baumer-Baumer-Mouradian, S.H.; Collins, S.; Lausten, T.; Pohl, C.; Sisney, M.; Khare, S.; Ose, M.; Roe, J.; Reilly, C.; Gutzeit, M., 2021 [37]	Urgent COVID-19 Vaccination of Healthcare Workers via a Quality Improvement Initiative	Cross-sectional	The article describes the development and implementation of a successful COVID-19 employee and community vaccination program. 3,921 healthcare workers completed the survey, and 73% reported intent to receive the COVID-19 vaccine immediately or later. After 57 clinic days, 83% (n = 5,231) of healthcare workers were vaccinated, and 99% completed the two-dose series. Vaccine waste was minimal at 0.1%.	Moderate confidence	The article highlights the unique differences between large-scale COVID-19 vaccination programs and established influenza vaccine campaigns. The authors failed to describe the method. They explain that a "vaccine team," including hospital leadership, employee health and wellness, nursing and pharmacy leadership, and a provider was formed to develop and implement a COVID-19 vaccination program. On the other hand, a survey was cited in the outcomes, which was not mentioned in the methodology. The study describes developing and implementing a successful COVID-19 employee and community vaccination program. The phenomenon and findings are coherent, and the qualitative and quantitative data are explained adequately. The children's health system is a freestanding, not-for-

					profit pediatric academic center and comprises a tertiary care hospital and primary and specialty pediatric care services. Therefore, the setting and population are relevant to the topic. The program was successful.
Li, M.; Luo, Y.; Watson, R.; Zheng, Y.; Ren, J.; Tang, J.; Chen, Y., 2021 [25]	Health care workers' (s) attitudes and related factors towards COVID-19 vaccination: a rapid systematic review	Systematic review	Vaccine acceptance varied widely and ranged from 27.7% to 77.3%. HCWs had positive attitudes towards future COVID-19 vaccines, while vaccine hesitancy was still common. Demographic variables such as male gender, older age, and medical profession were positive predictive factors. Women and nurses showed more vaccine hesitancy. Previous influenza vaccination and self- perceived risk were facilitators. Concerns for safety, efficacy, and effectiveness and distrust of the government were barriers. Influences of direct (COVID-19) patient care towards vaccination intention were less conclusive. Tailored communication strategies were needed to increase the uptake rate of COVID-19 vaccines among HCWs. More importantly, more data and information on the safety and efficacy of vaccines should be provided with transparency.	High confidence	There are minor methodological limitations to the review findings. This rapid systematic review has limitations since there was no search in the grey literature and only two languages were included in the criteria. The study is coherent regarding the target phenomenon, findings, and data. The study reports adequate data. The "herd immunity" concept is used, which was commonplace at the beginning of the COVID-19 pandemic. The study is relevant because the USA was an important country for assessing HCWs' willingness to be vaccinated. The results showed that HCWs had positive attitudes towards future COVID-19 vaccines, while vaccine hesitancy was still common. Demographic variables such as male gender, older age, and the medical profession were positive predictive factors. Women and nurses showed more vaccine hesitancy. Previous influenza vaccination and self- perceived risk were facilitators. Concerns for safety, efficacy, and effectiveness and distrust of the government were barriers. The influence on vaccination intention of having performed frontline COVID-19 patient care was less conclusive. The authors suggest a tailored communication strategy to increase COVID-19 vaccine uptake among HCWs.
